# Multi-Platform Metabolomic Analyses of Ergosterol-Induced Dynamic Changes in *Nicotiana tabacum* Cells

**DOI:** 10.1371/journal.pone.0087846

**Published:** 2014-01-31

**Authors:** Fidele Tugizimana, Paul A. Steenkamp, Lizelle A. Piater, Ian A. Dubery

**Affiliations:** 1 Department of Biochemistry, University of Johannesburg, Auckland Park, Johannesburg, South Africa; 2 Drug Discovery and Development, CSIR Biosciences, Pretoria, South Africa; RIKEN PSC, Japan

## Abstract

Metabolomics is providing new dimensions into understanding the intracellular adaptive responses in plants to external stimuli. In this study, a multi-technology-metabolomic approach was used to investigate the effect of the fungal sterol, ergosterol, on the metabolome of cultured tobacco cells. Cell suspensions were treated with different concentrations (0–1000 nM) of ergosterol and incubated for different time periods (0–24 h). Intracellular metabolites were extracted with two methods: a selective dispersive liquid-liquid micro-extraction and a general methanol extraction. Chromatographic techniques (GC-FID, GC-MS, GC×GC-TOF-MS, UHPLC-MS) and ^1^H NMR spectroscopy were used for quantitative and qualitative analyses. Multivariate data analyses (PCA and OPLS-DA models) were used to extract interpretable information from the multidimensional data generated from the analytical techniques. The results showed that ergosterol triggered differential changes in the metabolome of the cells, leading to variation in the biosynthesis of secondary metabolites. PCA scores plots revealed dose- and time-dependent metabolic variations, with optimal treatment conditions being found to be 300 nM ergosterol and an 18 h incubation period. The observed ergosterol-induced metabolic changes were correlated with changes in defence-related metabolites. The ‘defensome’ involved increases in terpenoid metabolites with five antimicrobial compounds (the bicyclic sesquiterpenoid phytoalexins: phytuberin, solavetivone, capsidiol, lubimin and rishitin) and other metabolites (abscisic acid and phytosterols) putatively identified. In addition, various phenylpropanoid precursors, cinnamic acid derivatives and - conjugates, coumarins and lignin monomers were annotated. These annotated metabolites revealed a dynamic reprogramming of metabolic networks that are functionally correlated, with a high complexity in their regulation.

## Introduction

Metabolomics is a holistic qualitative and quantitative analysis of all metabolites present within a biological system under specific conditions [1]–[4]. Metabolomics differs from the classical or traditional targeted phytochemical analysis in various fundamental aspects such as being a data-driven approach with predictive power that aims to assess all measurable metabolites without any pre-conception or pre-selection. In order to attain this goal, advanced analytical tools that provide high degrees of sensitivity, selectivity and reproducibility are required [3], [5]–[7]. Metabolomics is viewed as a complementary technique to other functional ‘-*omics’* approaches such as transcriptomics and proteomics. The integration of the these technologies contributes to a systems biology overview [8], providing a holistic understanding of the organisation principle of cellular functions at different levels, and ways of monitoring all biological processes operating as an integrated system [4], [9]–[11]. Moreover, metabolomics as a post-genomics tool is often regarded as offering distinct advantages when compared to other ‘omics’ technologies. This point of view is based on the fact that changes in the transcriptome or proteome do not always correlate to biochemical phenotypes [4], [7], [12], [13].

The holistic analysis of the metabolome, with its complex/divergent physico-chemical properties and dynamic molecular composition, requires a wide range of chemistries. Hence, all metabolomic analyses are like a snapshot (or point-in-time-chemistry) of a biological system (cell, tissue or whole organism), showing which metabolites are present and the levels at a given time point and under specific physiological conditions [1], [3], [7], [11], [14]. Different strategies and a range of analytical techniques have thus been developed for different metabolomic analyses; and the usage of parallel analytical platforms can provide a wide coverage of the metabolome under study, additional information or confirmation for a putatively identified metabolite [3], [14]–[16].

In plant research, metabolomic approaches are increasingly being used for various studies including linking genotype and biochemical phenotype, silent phenotypic mutations, metabolic pathway studies, and abiotic– and biotic stresses, including plant : pathogen interactions [2], [4], [9], [10], [13], [17]–[20]. Plants are continuously threatened by a wide range of pathogens or abiotic stresses and, for protection, all plants possess well-established resistance mechanisms developed through evolution. The innate immune system of plants can be divided into two fundamental components: protection and defence [21], [22]. Protection is a static (passive) phenomenon and involves both structural barriers and pre-formed inhibitors. These protective mechanisms prevent or attenuate invasion by potential attackers [21], [23]–[25]. On the other hand, defence is an inducible, dynamic (active) phenomenon and occurs only when the host and the pathogen have made physiological contact [21], [22]. The final outcome of the plant : pathogen interaction depends ultimately on the balance between the ability of the pathogen to suppress and overcome the plant’s immune responses, and the capacity of the plant to recognise the pathogen and activate effective defences [26], [27]. Recognition involves the binding of microbe-associated molecular pattern molecules (MAMPs) to pattern recognition receptors (PRRs) in the host. A detailed understanding of plant resistance mechanisms (protective and inducible response) opens up possibilities of developing strategies to increase stress/disease resistance in plants, noting that the latter are an essential part of human life (foods, medicines, industrial raw materials, *etc*.) and a vital component of the whole ecosystem.

Among the phytopathogens are fungi that continue to pose a significant threat to crop production and subsequently to food supply. The early stages of plant : pathogenic fungal interactions are mostly mediated by fungal MAMP molecules such as the evolutionary conserved ergosterol; a 5,7-diene sterol. Ergosterol does not occur naturally in plants and differs structurally from the phytosterols, having two additional double bonds (at positions C7–C8 and C22–C23) and a methyl group at C24 of the side chain. It is thus recognised by such cells as ‘non-self’ [28], thereby acting as a MAMP molecule in the lipophilic class of biotic elicitors [29]. The effect of ergosterol on plant secondary metabolism has not been thoroughly investigated [30]–[32]. Following our previous report [33], here we present a multi-platform metabolomics-based elucidation and analysis of changes in the metabolism of tobacco (*Nicotiana tabacum*) cells following ergosterol treatment. The results significantly contribute to a more comprehensive understanding of the defence-related phenotypic status of plants.

## Materials and Methods

### Chemicals and Reagents

All chemicals used were of analytical and ultra-pure LC-MS grade quality, and organic solvents mostly included chloroform (Labscan, Poland), methanol (Labscan, Poland) and acetone (Associated Chemical Enterprises, South Africa). All equipment was sterilised prior to use and cell treatment was carried out under sterile conditions.

### Cell Culture and Elicitation of Tobacco Cell Suspensions

Cell suspensions are used in metabolomics studies as systems that allow reproducibility and controllability of conditions [34]–[36], and *Nicotiana tabacum* cv. Samsun cell suspensions, cultivated as previously described [37], [38] were used. For elicitation of cells, a stock solution of 4.54 mM ergosterol, C_28_H_44_O, (Sigma, USA), was prepared in acetone. The final concentration of acetone in the treated suspensions did not exceed 0.05% and had no discernable effect on the observed responses.

Three days after subculture, cells were treated by adding specific volumes of the stock solution of ergosterol to aliquots of cells suspensions with continuous rotation at 80 rpm and 25°C. For concentration studies, ergosterol was added to final concentrations of 0–1000 nM, with an 18 h incubation period while non-treated cell suspensions were used as negative controls. For the time study, cell suspensions were treated with 300 nM ergosterol and incubated for 0–24 h with a non-treated sample, incubated for 24 h, included as a control. One of the key points in metabolomic studies lies in differentiating true biological variation from technical variation. Three independent biological repeats were therefore conducted and analysed.

### Cell Viability Determination

In order to determine if possible secondary responses due to treatment-induced cell death occurred, a cell viability assay was performed based on the reduction of 3,5-triphenyltetrazolium chloride (TTC) to red triphenylformazan (TPF) as previously described [33], [39].

### Metabolite Extraction

Extraction methods for metabolomic studies depend on the chemical and physical properties of the target metabolites, the biochemical composition of the system from which the analytes are to be extracted and the properties of the solvents to be used [15], [40]. The main extraction method developed to extract these metabolites was the dispersive liquid-liquid microextraction (DLLME) technique [41] using methanol and chloroform as disperser and extraction solvents respectively [33]. Briefly, DLLME was carried out as follows: after induction with ergosterol and incubation, the excess media was filtered using a Buchner funnel and the cells collected. Two grams of cells were re-suspended in 20 mL of methanol and homogenised using an Ultra Turrax homogenizer. The homogenates were centrifuged at 5525×*g* for 7 min at 25°C. The supernatants were placed into clean 50 mL round-bottom flasks, and the methanol evaporated to 1 mL at 50°C using a Buchi Rotavapor. One mL aliquots of the resulting crude aqueous extracts were transferred to glass centrifuge tubes and 200 µL of chloroform and 100 µL of methanol were added. The mixture was vortexed for 30 s and centrifuged at 9 000*×g* for 6 min at 25°C. Using a syringe, the bottom chloroform extraction layer was removed and filtered through 0.22 µm filters (Millipore, USA), and placed in glass vials. The chloroform extracts were kept at −20°C until further analysis.

A general methanol extraction was also used with the aim of extracting more polar metabolites. The first steps of this methanol extraction, from the induction to the Buchi Rotavapor methanol-evaporation step, were carried out as described above in the DLLME method. The 1 mL crude aqueous extract (following the evaporation of methanol) was placed into clean 2 mL microcentrifuge tubes and evaporated to dryness under vacuum using a SpeedVac centrifugal evaporator at 55°C. To the dry extract, 100 µL of methanol and 100 µL MilliQ water were added. The mixture was vortexed until all content was dissolved. The 50% methanol extracts were then filtered through 0.22 µm filters and placed into glass vials for chromatographic analyses. The methanol extracts were kept at −20°C until further analysis.

Following the extraction of the metabolites by the two methods, the extracts were further analysed, qualitatively and quantitatively, using various chromatographic techniques, mass spectrometry, and one-dimensional proton-nuclear magnetic resonance spectroscopy. Hence, to ensure that the data acquired from these analytical techniques were acceptable for further multivariate data analysis, three technical repeats for each biological repeat were analysed, for all experiments.

### ^1^H NMR Spectroscopy Analyses of Chloroform Extracts

One-dimensional proton nuclear magnetic resonance spectroscopy (1D ^1^H NMR) analyses were performed using “1PULSE” Fourier-transform (FT)-NMR experiments. All spectra were measured using a Bruker AVIII-400 NMR spectrometer, operating at a proton NMR frequency of 400.17 MHz at 296.8 K. For each sample, 64 scans (number of scans, NS) were recorded using a 9.30 µs (60°) pulse (P1, pulse width/pw), 8.22 kHz spectral width (SWH), and 1.00 s relaxation delay (D1/d1), and 0.125 Hz/point (FIDRES/res). The acquisition time (AQ/at) was set at 3.98 s and the free-induction decays (FIDs) were Fourier transformed with a line broadening (LB) factor of 0.30 Hz. For quantitative analysis, peak height was used. The CDCl_3_ solvent used contained the internal standard, trimethylsilyl propionate (TSP). The spectra were referenced to both residual solvent signal of CHCl_3_ (7.240 ppm) and TSP at 0.000 ppm.

### Gas Liquid Chromatography Coupled to Flame Ionisation Detection (GC-FID) and Electron-Impact-Mass Spectrometry (GC-EI-MS) Analyses

DLLME/chloroform extracts were analysed on a Shimadzu-17-10A GC instrument (Shimadzu, Kyoto, Japan), equipped with a 30 m×0.25 mm×0.25 µm Zebron ZB-1MS column (Phenomenex, California, USA). A 2 µL aliquot was injected into the GC-FID instrument in splitless mode. The injection and detector temperatures were set to 250°C and 350°C respectively. The overall programmed-temperature GC run included zones with different temperature gradient (multi-linear programming) with initial and final isothermal zones. The initial GC oven temperature was 80°C for 4 min, increased by 25°C min^−1^ to 240°C, then increased to 300°C at a rate of 5°C min^−1^ and held at 300°C for 3.60 min. The total run time was 26.00 min. The flow rate was set to 1 mL min^−1^, with nitrogen as the carrier gas. The detection mode in this GC approach was flame ionisation detection (FID). The solvent (chloroform) blank was injected after baking the column and cleaning the injection port on the GC instrument to determine if any peaks were attributable to the solvent (no significant peak was present apart from the solvent peak). This was done prior to the analysis of each biological replicate.

GC-MS was generally used for identification purposes as MS detection gives structural information about the analytes. In this experiment, a Shimadzu GC-MS-QP2010 (Shimadzu, Kyoto, Japan) with an electron impact (EI) ion source and a quadrupole mass analyser was employed. The column used was a ZB1-MS non-polar column (Phenomenex, California, USA). GC conditions/parameters were set as described above. The injection mode was set to a splitless; and 1 µL of the chloroform sample was manually injected. The MS was operated using a positive EI at 70 eV. Helium was used as the carrier gas, and the column flow rate set at 1 mL min^−1^. The ion source temperature was set at 200°C, and the detector voltage was set at 0.5 kV to prevent the oversaturation of the detector since the chloroform extracts were concentrated. The solvent cut time for the MS was set at 5 min and EI scan mode was used for identification covering the range of 50–500 *m/z*.

### Comprehensive 2D GC Coupled to TOF-Mass Spectrometry Analyses

In the GC×GC technique the two columns are coupled by a special interface (modulator) that is capable of sampling the effluent from the first column (first dimension) and periodically introducing it to the second column (second dimension) in a manner that preserves the original first dimension separation [42]–[44]. The DLLME chloroform extracts were analysed using a Pegasus 4D GC×GC-TOF-MS instrument from LECO Corporation (St. Joseph, MI, USA). Samples were run in two-dimensional mode. The sample injection volume was 1 µL with helium as the carrier gas at a flow rate of 1.00 mL min^−1^. The inlet temperature was set at 250°C and a splitless mode injection type was used. The first dimension column was a mid-polar Restek Rxi-17 S matrix column (15 m, 360 µm i.d., 0.25 µm d.f.) and the temperature profile was 10.00°C min^−1^ from 50 to 325.00°C with a hold for 1.00 min at 50°C and for 10.00 min at 325.00°C. The second dimension column was a non-polar Restek Rtx-5 matrix column (0.990 m, 180 µm i.d., 0.20 µm d.f.) and the temperature profile was 10.00°C min^−1^ from 65 to 340°C with a hold for 1.00 min at 65°C and for 3.00 min at 340°C. The first and second dimensional columns were linked by a modulator, and the modulation timing was set at 5.00 s. The detector was a time-of-flight (TOF)-mass spectrometer, and electron impact (EI) was used as the ionisation mode. The ion source temperature was set at 200°C while the detector voltage was set at 1750 V and the electron energy at −70 V. The mass range collected was 40–550 Da with an acquisition rate of 100 spectra s^−1^.

### Ultra High Performance Liquid Chromatography-High Definition Mass Spectrometry (UHPLC-HDMS) Analyses

UHPLC and High Definition Mass Spectrometry (UHPLC-HDMS) analyses were performed on a Waters Acquity UHPLC coupled in tandem to a Waters photodiode array (PDA) detector and a SYNAPT G1 HDMS QTOF mass spectrometer (Waters, Manchester, UK). The combination of UHPLC with a PDA and QTOF detectors allow for the tentative identification of compound classes, and the possibility to obtain a more conclusive identification of separated compounds utilising mass spectrometric measurements and software tools. The HDMS technique (Waters, Manchester, UK) provides much structural information, and particularly high mass measurement accuracy, typically better than 5 mDa, which is a key factor in compound identification in metabolomics. This HDMS technology expands beyond the conventional MS due to its capabilities such as extra-dimensional high efficiency ion mobility, improved analytical peak capacity and selectivity, enhanced sensitivity, efficient sampling of ions, and elimination of neutral contaminants [45]–[47].

Mass accuracy was obtained by calibration of the instrument over the mass range of the analytical method and was further enhanced by the use of a reference mass channel generated by the use of a lock spray interface and leucine enkephalin as calibrant. Any fluctuations in the accuracy of mass measurements, caused by temperature fluctuations and electronic noise, were automatically corrected if needed. This design allowed for extended run times as well as batch analysis spreading over several days without compromising mass accuracy.

Chromatographic separation of both the chloroform and methanol extracts was done utilising a Waters CSH C18 Acquity column (150 mm×2.1 mm, 1.7 µm) thermostatted at 60°C. A binary solvent mixture was used consisting of water (eluent A) containing 10 mM formic acid (pH of 2.3) and acetonitrile (Romil pure chemistry, UK) (eluent B). The initial conditions were 95% A at a flow rate of 0.4 mL min^−1^ and were kept constant for 2 min. A gradient was introduced to change the chromatographic conditions to 5% A at 22 min. The conditions were kept constant for 3 min to flush the analytical column whereafter the column was returned to initial conditions at 27 min and allowed to equilibrate for 3 min. The run time was 30 min and the injection volume was 5 µl. Each sample was analysed in triplicate, to account for any analytical variability. The PDA detector was scanned between 200 and 500 nm (1.2 nm resolution) and collecting 20 spectra s^−1^.

The SYNAPT G1 mass spectrometer was used in V-optics and operated in electrospray ionisation (ESI) mode to detect the compounds of interest. Leucine enkephalin (50 pg mL^−1^) was used as reference calibrant to obtain typical mass accuracies between 1 and 3 mDa. The mass spectrometer was operated in both positive and negative mode with a capillary voltage of 2.5 kV, the sampling cone at 17 V and the extraction cone at 4 V. The scan time was 0.1 s covering the 100–1000 Da mass range. The source temperature was 120°C and the desolvation temperature was set at 450°C. Nitrogen gas was used as the nebulisation gas at a flow rate of 800 L h^−1^. The software used to control the hyphenated system and perform all data manipulation was MassLynx™ 4.1 (Waters Corporation, USA).

### Multivariate Data Analysis

In comparison to traditional univariate statistical methods, the MVDA models are well suited to provide ways of handling confounding and covariance patterns (both within and between variables), which are found in complex and multi-dimensional data sets from metabolomics studies [48], [49]. Principal component analysis (PCA), an unsupervised multivariate linear model, and orthogonal projection to latent structures-discriminant analysis (OPLS-DA), a supervised model, were used for data analysis. Only GC-FID, UHPLC-MS and NMR data were MVDA-modelled, generating sufficient and conclusive information which was supplemented by visual inspection of chromatograms and NMR spectra.

The quality of the models was evaluated based on some model diagnostic tools largely used in metabolomic studies, namely the cumulative modelled variation in matrix X, *R^2^X*(cum) or the goodness-of-fit parameter, the proportion of the variance of the response variable that is explained by the model, *R^2^Y*(cum), and the fraction of the total variation of matrix X that can be predicted by the extracted components, *Q^2^*(cum) known also as predictive ability parameter. The values of these diagnostic parameters must be close to 1.0 for a robust mathematical model with a reliable predictive accuracy [50], [51].

### ^1^H NMR Data Analysis

^1^H NMR spectra were automatically reduced to ASCII files using AMIX software (version 3.7, Bruker Biospin, Germany). Spectral intensities were scaled to total intensity and reduced to integrated regions of equal width (0.04 ppm) corresponding to the region of δ 0.1–10.00. The region of δ 7.3 was excluded from the analysis because of the residual signal of chloroform. The data matrix obtained was exported to the SIMCA-P software version 13.0 (Umetrics, Umea, Sweden) for PCA modelling using the *Pareto* scaling method. The *Pareto* scaling involves dividing the variables by the square root of their standard deviations [49], [52]–[54]. PCA scores and loadings plots were used to explain variations in the samples.

### GC-FID Data Analysis

For GC-FID data analysis, a data matrix was generated from the retention time (Rt) and peak area chromatographic data (from the Shimadzu-17-10A GC instrument). This dataset was exported into the SIMCA-P software version 13.0 (Umetrics, Umea, Sweden) for PCA modelling. The PCA scores plot was used to depict a visual image of sample variations from a global view. The data were all *Pareto*-scaled so as to reduce the impact of noise and artefacts in the models, improving subsequently the models’ predictive ability.

### UHPLC-MS Data Analysis

For UHPLC-ESI-MS data, both PCA and OPLS-DA models were used. ESI positive and negative raw data were extracted using MassLynx™ XS software and analysed with MarkerLynx™ XS software (Waters Corporation, Mildford USA). The MarkerLynx software extracts the raw LC-MS data and produces a matrix of Rt-*m/z* variable pairs, with the *m/z* peak intensity for each sample. MarkerLynx software parameters were set to analyse the 2–26 min Rt range of the chromatogram, mass range 100–700 Da, mass tolerance 0.01 Da, mass window 0.05 Da and a Rt window of 0.20 min. The data matrix obtained from MarkerLynx processing was also exported to the SIMCA-P software for PCA and OPLS-DA modelling. In both models, the data were *Pareto*-scaled. PCA scores and loadings plots were used to explain variations in the samples. The OPLS-DA S-plot aided in biomarker/compound identification. The S-plot helps in explaining and visualising the covariance and correlation between ions and the modelled classes, allowing thus the mining of the data to extract metabolites that are statistically interesting compounds with potential biochemical significance [54]. Furthermore, the significance of the selected mass ions (variables) in OPLS-DA models was also assessed using the variable importance in projection, (VIP) plot (Umetrics, Umea, Sweden).

### Metabolite Annotation

#### GC-MS

For GC-EI-MS the respective mass spectra of the extracted ion peaks identified by comparison of the total ion current (TIC) mass chromatograms were searched against the NIST (National Institute of Standards and Technology) and Wiley mass spectra libraries (2007 versions) for similarity matches. This spectral comparison provided putative empirical formulae and structures, which were further searched in databases such as the Dictionary of Natural Products (DNP) (www.dnp.chemnetbase.com) and ChemSpider (www.chemspider.com). For GC×GC-TOF-MS analyses, the Chroma-TOF software (LECO Corporation, St. Joseph, MI, USA) was used for automated data analysis from preprocessing to the compound identification step.

#### UHPLC-MS

Selected mass ions from S- and VIP plots were annotated using the Taverna workbench for PUTMEDID_LCMS Metabolite ID Workflows (www.taverna.org.uk). The Taverna workflows allow for integrated, automated and high-throughput annotation and putative metabolite identification from ESI-LC-MS metabolic data. The workflows consist of correlation analysis, metabolic feature annotation and metabolite annotation. MarkerLynx XS data were exported into the Taverna workflows to be processed for metabolite annotation. The data matrix from MarkerLynx processing was firstly formatted to match the Taverna workbench requirements. Three main workflows formed the Taverna metabolite annotation procedure. In the workflow 1 (*List_CorrData*), all the parameters were set at default, ion mode selected (as negative or positive depending on the ion mode in which the data where acquired) and Pearson correlation calculation was chosen as the correlation method. In the workflow 2 (*annotate_Massmatch*), mass tolerance was set to 5 ppm, retention time range set as 120–1560 s (2–26 min) and all other parameters were set at default. These workflows allowed for grouping together ion peaks with similar features such as Rt, and annotating features with the type of *m/z* ion (molecular ion, isotope, adduct, others) believed to originate from the same compound. The elemental composition/molecular formula (MF) of each *m/z* ion is then automatically calculated. In workflow 3 (*matchMF-MF*) the calculated MF (from the workflow 2 output file) was then automatically compared and matched to the MF from a pre-defined reference file of metabolites (a customised chemical library based mainly on PlantCYC and AraCYC libraries). The selected MF were also manually searched against freely online databases such as DNP, Chemspider and METLIN libraries for more confidence in the annotated metabolites (MI-level 2 annotation).

## Results and Discussion

The triphenyltetrazolium chloride-based cell viability assay [39], was performed to determine if treatment-induced cell death occurred under the experimental conditions. The results (not shown) indicated that treatment of the cell suspensions with ergosterol (0–1000 nM) did not lead to a significant cell death and that >91% of cells remained viable as estimated at 1000 nM, incubated for 18 h. The observed responses are thus due to the ergosterol treatment alone and possible secondary responses due to cell death can be excluded.

In order to investigate the ergosterol-induced changes in the intracellular metabolome (as present in both semi-selective DLLME chloroform extracts and a general methanol extraction (ME) of tobacco cells, concentration- and time studies were conducted. These investigations served also to establish the optimal conditions for treatment of cells, which were found to be 300 nM ergosterol and an 18 h incubation period as discussed in this section. One-dimensional proton NMR spectroscopy and different chromatographic techniques (GC-FID, GC×GC-TOF-MS, UHPLC-MS) were used for the analyses. The combination of these analytical platforms provided a broader and more conclusive observation of the effect of ergosterol treatment on the metabolism of tobacco cells.

### One-Dimensional Nuclear Magnetic Resonance (1D ^1^H NMR) Spectroscopic Analyses

Chloroform extracts, obtained through the DLLME procedure, were analysed by 1D ^1^H NMR to investigate metabolite variations due to different ergosterol concentration treatments. The NMR technology has been widely used as a fingerprinting tool in metabolomic analyses, with pattern recognition techniques such as PCA modelling [35], [55], [56]. NMR is a non-biased, non-destructive technique and one of the few analytical platforms that are rapid, reproducible, stable and require very little or no sample preparation [1], [5], [57]. A ^1^H NMR spectrum of a plant extract is the result of the superposition of the ^1^H NMR spectra of all NMR-visible single compounds present therein. Hence, a proton NMR analysis would give a global view of all the metabolites (primary and secondary) in a sample, provided there are ^1^H NMR detectable [5], [57].

**Figure 1:**
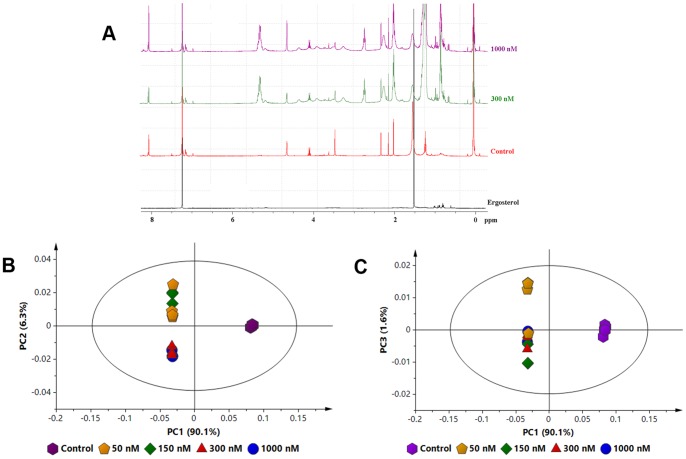
Proton NMR spectra of extracts from ergosterol-treated cells. (**A**) Overlaid ^1^H NMR spectra of DLLME samples representative of control, 300 nM and 1000 nM ergosterol-treated tobacco cells showing differences in proton shifts/signals due to ergosterol-induced changes across the 1–8 ppm region of the spectrum. The ^1^H NMR spectrum of ergosterol is at the bottom. Deuterated chloroform was used as the solvent; however, since deuteration is not 100%, the residual protons from chloroform (CHCl_3_) give a singlet signal at 7.26 ppm. (**B**) and (**C**) PCA scores plots of the ^1^H NMR data from the concentration study. DLLME extracts of the cells treated with 0 (control), 50, 150, 300 and 1000 nM ergosterol, and incubated for 18 h. The scores plots shows the clustering/separation of different treatments with little variation within each group.

Here, the DLLME samples were analysed using 1D ^1^H NMR spectroscopy since the aim was to generate a global view or a fingerprint of the effect of different ergosterol concentrations on tobacco cells from a ^1^H NMR perspective. Figure 1 represents the results obtained from the 1D ^1^H NMR analyses of the DLLME concentration study samples.

The visual inspection of the overlaid NMR spectra of ergosterol-treated and non-treated control samples (Figure 1A) show treatment-related differences in various spectral regions: δ0-δ3, δ3-δ5.8 and δ5.8-δ10. These spectral regions are often known to be associated with amino acids, sugars and aromatic compounds, respectively [57]. However, other compounds also resonate in these regions. For instance, fatty acids and terpenes resonate in the 0–3 ppm region and the sesquiterpenoids, rishtin and capidiol, also resonate in the 3–5.8 ppm spectral region [58].

The obtained NMR data were analysed by unsupervised MVDA modelling, PCA, to differentiate between ergosterol treatments, and to provide interpretable visualisation of the NMR spectra. PCA reduces the dimensionality of the data without much loss of information, and expresses the data in such a way as to identify and highlight the similarities and differences in systematic patterns and features of the data set. The PCA scores plot offers a visual image of sample variations from a global view and, being a non-parametric analysis, the generated model is independent of the user, hence unsupervised [48], [49], [59]. A five-component model, explaining 99.6% of the variance (with the accuracy of prediction of 0.991), was calculated. Scores plots PC1 vs. PC2 [*R^2^X*(cum) of 0.98, *Q^2^*(cum) of 0.969 and 95% confidence] and PC1 vs. PC3 [*R^2^X*(cum) of 0.91, *Q^2^*(cum) of 0.947 and 95% confidence] were constructed (Figures 1-B and -C), showing a differential clustering of the samples. The non-treated samples appear separate from the ergosterol-treated samples. The samples treated with 300 nM and 1000 nM ergosterol grouped together, with less variation between these two clusters.

**Figure 2:**
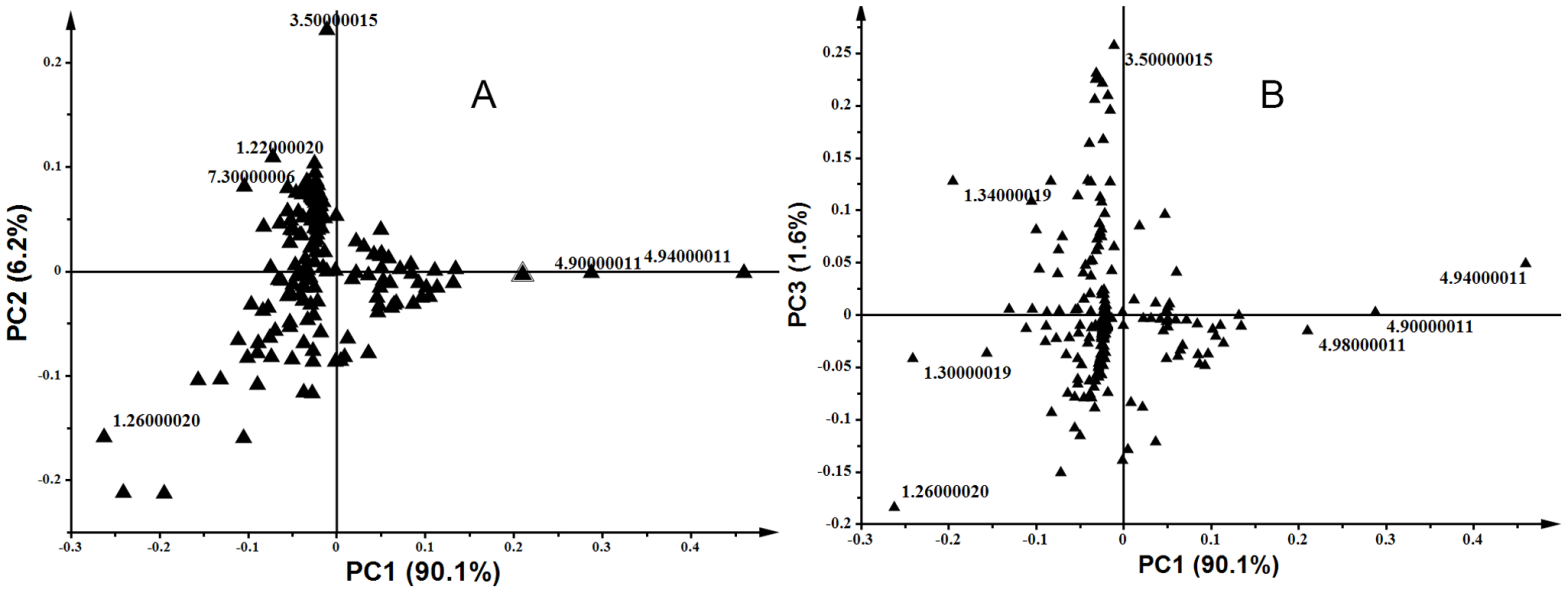
Principal component analysis (PCA) loadings plots of ^1^H NMR spectral data. DLLME extracts were prepared from cells treated with 0 (control), 50, 150, 300 and 1000 nM ergosterol, and incubated for 18 h. The loadings plot shows the discriminating variables responsible for sample clustering depicted by the scores plots in Fig. 1 (B and C). (**A**) PC1 vs. PC2 and (**B**) PC1 vs. PC3.

This NMR-based global view, as depicted by the PCA scores plots (Figure 1-B and -C), demonstrates that ergosterol treatment led to differential changes in the metabolite composition of the tobacco cells. The loadings plots (Figure 2) single out the discriminating variables responsible for sample clustering to be putatively compounds resonating in the 3–5.8 ppm spectral region (δ3.5 and δ4.9 chemical shifts), and compounds abundantly in the amino acid/fatty acid spectral region (δ0-δ3 chemical shifts). Considering the low polarity of chloroform as solvent, it could be possible that the chemical shifts that contributed to the sample clustering are from sesquiterpenoids (in the 3–5.8 ppm spectral region) [58], fatty acids (in 0–3 ppm spectral region) and related precursor molecules [60]–[63]. However, no further definitive chemical elucidation of these NMR-detected compounds was carried out, which might require 2D NMR analyses to resolve any overlapping signals, or hyphenating NMR and LC to introduce a separation dimension [5], [57]. Here, however, the NMR global view was sufficient to demonstrate ergosterol-induced metabolic changes. Furthermore, the inherent low sensitivity of NMR, its sensitivity to the chemical environment (pH, ionic strength, temperature, etc.) of the sample and the differential sensitivity of metabolites to the chemical environment hamper the quality of NMR analyses of complex samples [57].

### Gas Chromatography-Flame Ionisation Detector (GC-FID) Analyses

**Figure 3:**
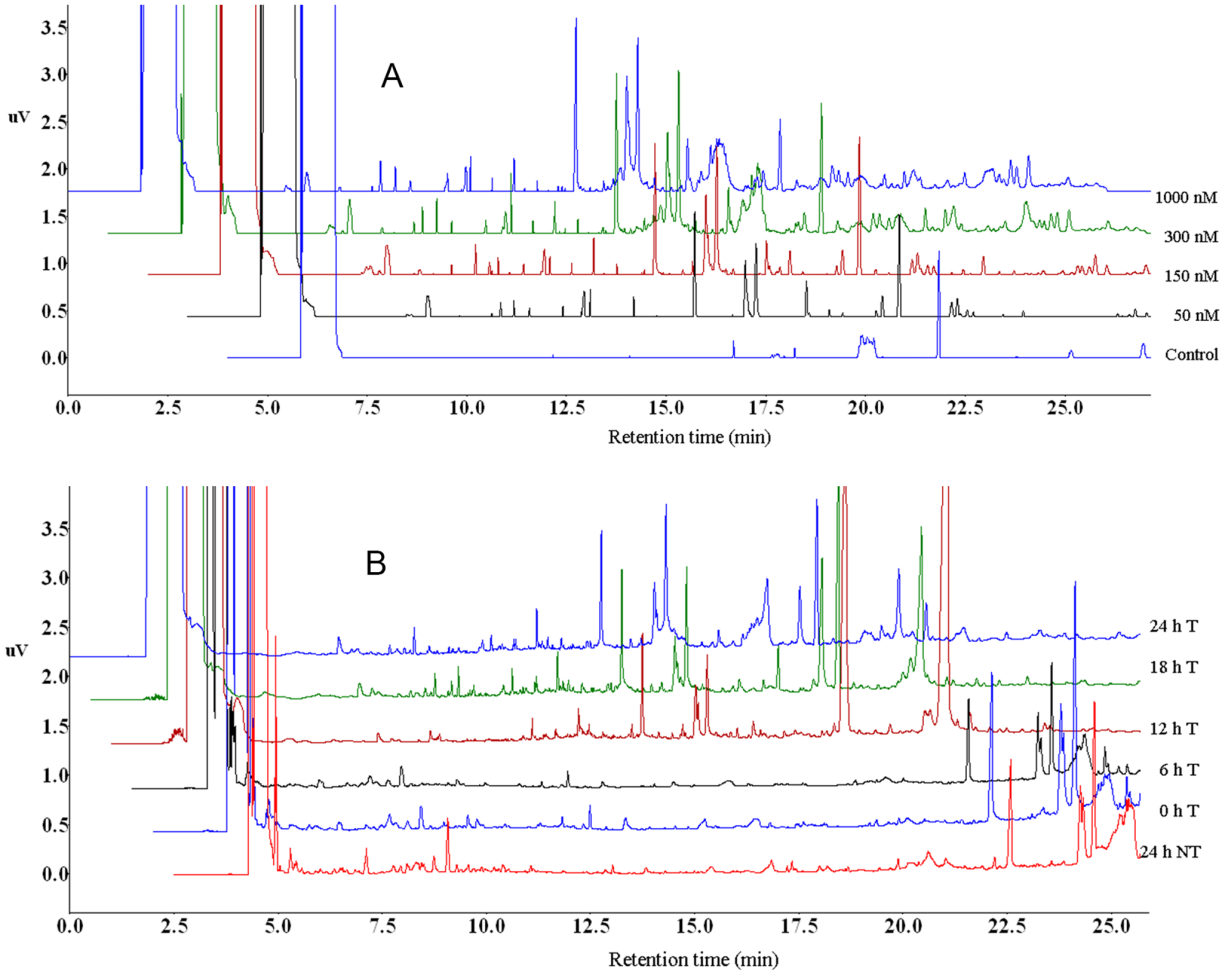
GC-FID chromatograms of DLLME extracts of ergosterol-treated (T) tobacco cells. (**A**) Chromatograms showing the differences between the control/non-treated cells (0 nM) and ergosterol-treated cells (50–1000 nM) incubated for 18 h. From bottom to top: 0 (control), 50, 150, 300 and 1000 nM. (**B**) Chromatograms showing time-dependent variations of extracts of tobacco cells treated with 300 nM ergosterol and incubated for different time periods (0, 6, 12, 18 and 24 h T). The bottom chromatogram is a non-treated (NT) sample incubated for 24 h (24 h NT).

Chloroform extracts, obtained through the DLLME procedure were analysed by GC-FID to investigate metabolite variations due to different ergosterol treatment conditions with regard to concentration and elicitation time. The samples were analysed in multi-linear programmed-temperature conditions to obtain a greater chromatographic separation. The resulting chromatograms (Figures 3-A and -B) showed concentration- and time-dependent variations in terms of peak intensities and presence/absence of peaks. The raw data were then preprocessed (peak alignment) and pre-treated (defining selected peaks) for further data analyses.

The GC-FID chromatograms (Figures 3-A and -B) indicate that various compounds in the DLLME samples were separated and detected, showing treatment-related variations of metabolites with low polarity and molecular weight [64], thus indicating changes in metabolite levels and expression, *i.e*. an altered metabolome.

**Figure 4:**
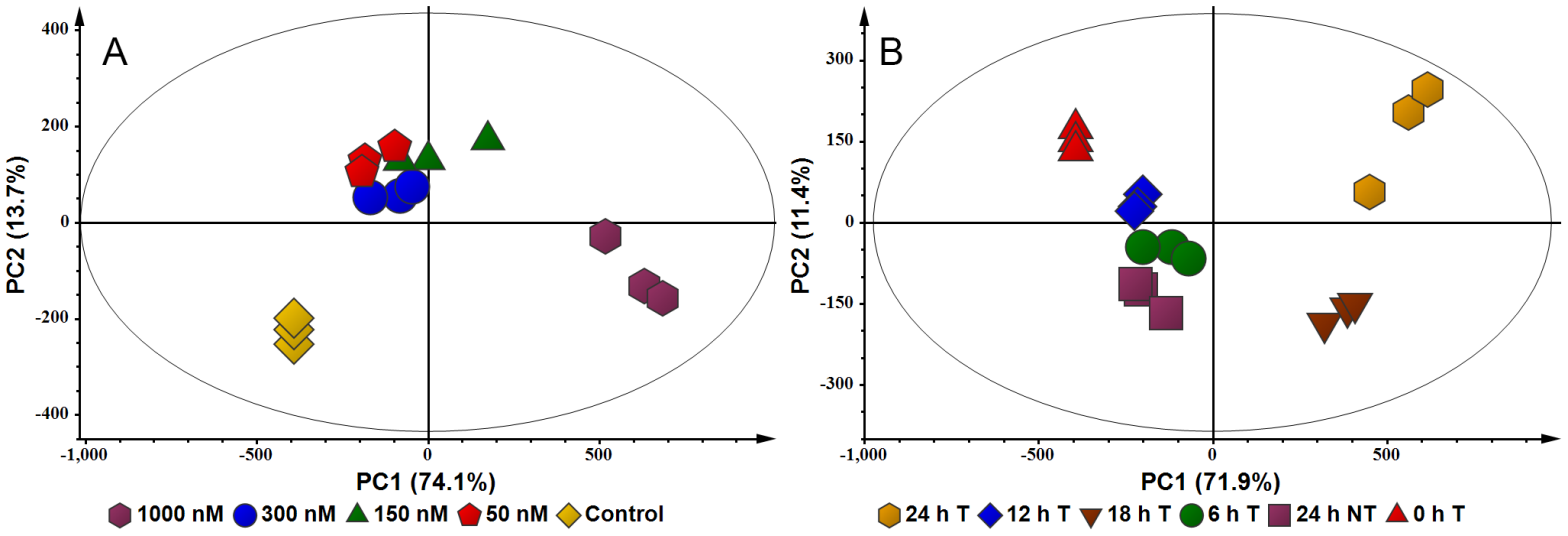
PCA scores plots of the GC-FID concentration- and time study data. (**A**) DLLME extracts of cells treated with 0 (control), 50, 150, 300 and 1000 nM ergosterol, and incubated for 18 h. The scores plot shows the clustering of different treatments (0–1000 nM) with little variation within each group. The 50–300 nM clusters appear to group together. The 0 nM (control) samples are clearly separated from the treated samples. (**B**) DLLME extracts of cells treated with 300 nM ergosterol and incubated for different time periods (0–24 h T) and non-treated samples incubated for 24 h (24 h NT). The scores plot shows the clustering of the different treatments, with the 18 h- and 24 h-treated samples being significantly separated from the other samples.

PCA was carried out to differentiate between ergosterol treatments. For the concentration study, a four-component model was generated and explained 94.7% of the variance [*R^2^X*(cum) of 0.947 and *Q^2^*(cum) of 0.810], with the first two principal components (PC1 and PC2) explaining 87.8%. A scores plot was constructed using PC1 and PC2 [*R^2^X*(cum) of 0.878 and *Q^2^*(cum) of 0.804; with 95% confidence level], showing cellular samples differentially clustered into five groups (Figure 4A). The clusters of 50-, 150- and 300 nM ergosterol-treated samples are found to be grouped together, which implies a similarity in the GC-FID detected metabolite composition of these samples. The non-treated (controls) and the 1000 nM ergosterol-treated samples are significantly separated from the other samples (*i.e*. different in metabolite composition).

For the time study data, a three-component model was calculated and explained 89.6% variation [*R^2^X*(cum) of 0.896 and *Q^2^*(cum) of 0.814]. The first two components (PC1 and PC2) explained 83.3% of the variance and were used to construct a scores plot [*R^2^X*(cum) of 0.833 and *Q^2^*(cum) of 0.742; with 95% confidence], showing samples differentially clustered into different groups (Figure 4B). Although treatment-related clusters formed, the 0 h treated samples were separated from the rest of the samples, while the non-treated samples (incubated for 24 h to compensate for aging of cells) were seen to group with the 6- and 12 h-treated samples, and the 18 h- and 24 h-incubated samples were clustered together, indicating that the biggest changes occurred in the period 12–18 h, and that the response was essentially complete at 18 h.

The GC-FID results thus reveal that ergosterol induces changes in DLLME-extractable secondary metabolites of tobacco cells. These changes include variation in the levels of the constitutively expressed metabolites and production of new metabolites, as demonstrated in Figures 3-A and -B. Differential changes in the intracellular metabolite profiles, explained by PCA (Figure 4), reflect the cells’ response to the perception of ergosterol.

Although the multi-linear programmed-temperature conditions in GC analyses improves the separation of the components in a sample [65], [66], the ultracomplexity/multi-dimensionality of the extracted plant metabolites makes baseline separation difficult [3], [67]. Overlapping peaks or co-eluting compounds that exist in the obtained chromatogram, can contribute to the observed variations. Furthermore, technical and instrumental variation can also contribute to minor variations in the chromatograms and thus influence the MVDA output [66]. Thus, to add a different dimension, DLLME samples were further analysed with a comprehensive GC×GC-TOF-MS technique.

### Two-Dimensional Gas Chromatography (GC×GC-TOF-MS) Analyses

**Figure 5:**
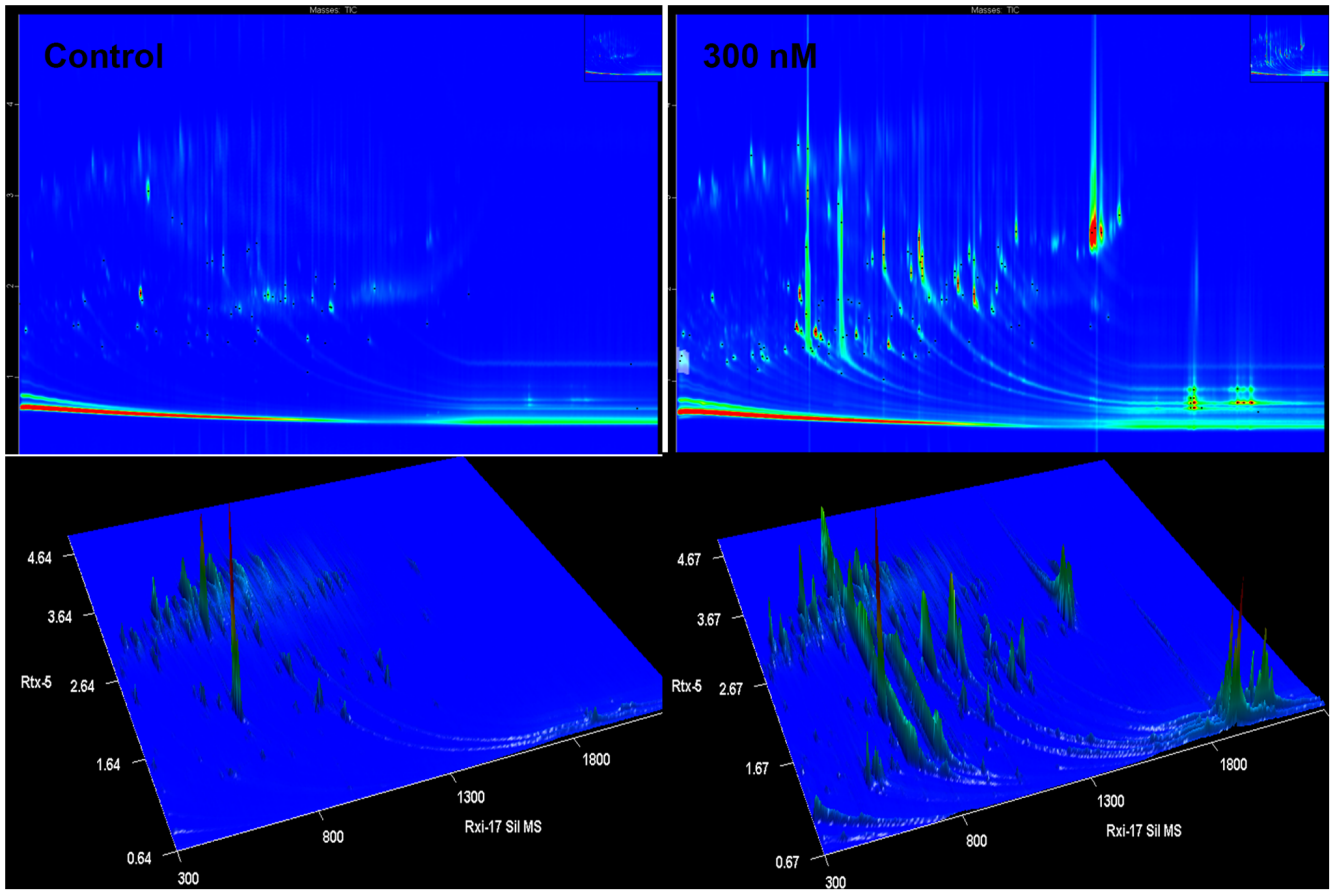
Representative 2D and 3D TIC chromatograms from the GC×GC-TOF-MS analyses. The representative 2D/3D TIC chromatograms of the DLLME samples show differences in terms of the number of ion peaks detected and the variation in peak intensities between non-treated and 300 nM ergosterol-treated samples, incubated for a period of 18 h.

The advantage of the GC×GC-TOF-MS technology is its multi-dimensional separation, which is based on the orthogonality of two columns that are used in this technique [42]. GC×GC-TOF-MS permitted an improved chromatographic separation of the DLLME samples for both the time and concentration studies, compared to the one-dimensional GC-FID analyses by providing an expanded separation space and therefore minimising possible co-elution of analytes or 1-D peak overlap. Figure 5 is a representative of the TIC chromatograms obtained from the GC×GC-TOF-MS analyses. Generally, each spot/dot in the 2D/3D-TIC chromatogram represents a detected ion peak. The visual inspection of these TIC chromatograms shows clear differences (in terms of the number/intensity of detected ion peaks) between control (non-treated) and ergosterol-treated samples.

Although the DLLME method selectively extracts semi- and non-polar compounds, the resulting analytes represent only part of the total metabolome. DLLME extracts are still multi-dimensional samples due to the inherent ultracomplexity of the plant metabolome. Due to differential and multiple decoration of a common skeleton (by methylation, prenylation, hydroxylation, conjugation, or acylation reactions), plant secondary metabolites are structurally highly diverse, forming a highly complex spectrum of compounds of different size, solubility, volatility, polarity, quantity and stability [4], [15], [63], [68].

Sample dimensionality is a measure of sample complexity and strongly influences component resolution in relationship to the dimensionality of the separation system [69]. Sample dimensionality, denoted as *s*, is an intrinsic property of analytical samples (other than the number of constituents) that determines their amenability to separation systems. This parameter *s* is defined as the number of independent variables that must be specified to identify the components of the sample, and it arises mostly from the physico-chemical differences of the sample constituents [69], [70]. Semi- and non-polar plant metabolites present in the DLLME extracts would come from different chemical families, with diverse decoration, forming a multi-dimensional sample. Multi-dimensional and/or hyphenated separation systems would ideally provide a better separation of such complex samples compared to one-dimensional system, such as 1D GC-FID [69]–[72].

The use of a GC×GC-TOF-MS in this study thus provided an enhanced peak capacity for gas chromatographic analyses of the DLLME extracts, and also revealed the internal structure of the sample constituents as represented by the 2D pattern of peak distribution in TIC chromatograms (Figure 5). The peak pattern generated from a GC×GC analysis can be used as defining the 2D signature of a sample’s components [73] and can be useful for a metabolic fingerprinting study. The visual inspection of the obtained chromatograms was sufficient to provide a level of analytical certainty regarding the ergosterol-induced variations, which were already observed from 1D GC-FID data. The MS-based annotation of compounds was obtained from the GC×GC-TOF-MS analyses of DLLME samples and is presented below.

Generally, only relatively small molecules of low polarity can be directly analysed by GC; hence the ergosterol-induced variations depicted by the GC analyses is, to a certain degree, limited by the GC capability. Liquid chromatography, on the other hand, permits separation of compounds of a wide range of polarity and molecular weight [64], [74], [75]. Thus, to obtain an even more comprehensive view of the effect of ergosterol on the metabolome, LC coupled to high definition mass spectrometry (UHPLC-HDMS) technology was subsequently employed as analytical technique. Aspects of the UHPLC-HDMS analyses of DLLME samples was previously reported, and showed that ergosterol triggered differential changes in the non−/semi-polar metabolome of tobacco cells, with the *de novo* biosynthesis of five bicyclic sesquiterpenoids phytoalexins: capsidiol, lubimin, rishitin, solavetivone and phytuberin [33]. The LC analyses of methanol extracted (ME) samples in this report also indicated differential metabolic changes related to ergosterol treatment (Figures S1–S3).

Considering the analytical range of GC and LC, the results from both GC and LC analyses evidently suggest that the defence response induced by ergosterol in tobacco cells comprises a metabolic reprogramming that involves a wide range of intracellular secondary metabolites (from thermally stable non-polar metabolites to the thermally labile, semi-polar metabolites, with different molecular weights and chemical structures). Due to the ultracomplexity of the plant metabolome and the inherent limitations of the existing technologies, no single analytical approach is entirely competent in fully covering the whole metabolome or a specific metabolic range within a given biological sample [3]–[5], [15]. Thus, the combination of 1D ^1^H NMR, GC-FID, GC×GC-TOF-MS and UHPLC-MS technologies and two different extraction methods (a selective DLLME and general ME) provided a relatively comprehensive snapshot of the effect of ergosterol on the metabolome of tobacco cells. In order to elucidate clear, specific biological insights into the effect of ergosterol on plant metabolism, the study further translated the observed ergosterol-induced metabolic changes (demonstrated by visual inspection of chromatograms/NMR-spectra and PCA-explained variations), into the annotation of known metabolites.

### Metabolite Annotation

Metabolite annotation or tentative/putative identification is one of the essential steps in metabolomic analyses, since biochemical interpretation of metabolomic data relies on the chemically annotated molecules from spectrometric/spectroscopic signals [12], [14]. However, one of the difficulties that arise in metabolomic studies is the identification of the *de novo*-induced compounds due to both the very restricted amounts and the high complexity of the biological extracts [76]. Furthermore, in the case of plant : pathogen interaction studies, the induced metabolites have varying rates of accumulation, and most of these metabolites may not be stable and can undergo degradation either by the plant or in the extraction process. In this regard, plants contain enzymes that degrade or convert antimicrobial compounds, returning their levels to pre-infection or pre-stress concentrations after the infection or stress has been contained or accommodated [77]. Thus, mass spectral data (from GC-EI-MS, GC×GC-TOF-MS and UHPLC-HDMS) were used for identification of biomarkers and *de novo* metabolites related to the observed ergosterol-induced changes in the tobacco cells.

The mass spectral data provide a pattern that is often compound specific. However, the degree of certainty in elucidating structural and chemical identity of an MS-detected analyte relies on the efficiency and accuracy of the three principal processes of the MS (ionisation, *m/z* analysis and ion detection) and on appropriate algorithms. The usage of parallel analytical platforms can provide additional information or confirmation for a putatively identified metabolite [11], [12], [78], [79]. Here, accurate mass measurement, high mass spectrum match factors, accuracy in calculating elemental composition, usage of some MS^E^ information (where available) and confirmation from parallel analytical platforms provided the certitude in metabolite assignments/annotations, thus indicated as ‘tentative’ or metabolite identification (MI)-level 2 [80].

### GC-EI-MS and GC×GC-TOF-MS Based Annotation of Non-polar, Volatile Compounds

For GC-EI-MS analysis, the search results against the NIST and Wiley mass spectra libraries were evaluated based on the probability factor, a match factor provided by the search algorithms that describes how unique a spectrum is, compared to all other spectra in the library [81]–[83]. Only spectra with probability hits in the range from 650 to 900 or above, were considered. Six metabolites (terpenoids and fatty acids) were thus annotated from the GC-EI-MS analyses of DLLME samples (Table 1 and Table S1).

**Table 1:** Some of the annotated/tentatively identified (MI-level 2) metabolites (with VIP scores >1.0) from ergosterol-treated tobacco cells.

#	*m/z*	Rt (s)	MF	MW(ave)	Compound ID name	Platform*	Metabolite category
**1**	265.1545	599.4	C_15_ H_24_O	220.35	Deoxy-capsidiol	LC-MS	Terpenoid
**2**	236	990; 1.79	C_15_O_24_O_2_	236	Capsidiol	LC-MS; 2D GC-MS	Terpenoid**
**3**	204	985; 1.65	C_15_H_24_	204	epi-Aristolochene	LC-MS; 2D GC-MS	Terpenoid
**4**	222	1009; 1.81	C_15_H_26_O	222	Farnesol	1D GC-MS;2D GC-MS	Terpenoid
**5**	155.1427	745.8	C_10_H_18_O	154.249	Geraniol	LC-MS	Terpenoid
**6**	449.1883	616.2	C_20_H_36_O_7_P_2_	450.443	Geranylgeranyl diphosphate	LC-MS	Terpenoid
**7**	291.1369	595.8	C_15_H_26_O_3_	254.365	Hydroxy-dihydrolubimin	LC-MS	Terpenoid
**8**	297.144	185.4	C_15_H_24_O_3_	252.349	Hydroxylubimin	LC-MS	Terpenoid
**9**	311.1166	642.6	C_15_H_24_O_2_	236.349	Lubimin	LC-MS	Terpenoid
**10**	261.1245	175.2	C_14_H_22_O_2_	222.323	Rishitin	LC-MS; 1D, 2D GC-MS	Terpenoid**
**11**	309.144	444	C_15_H_22_O	218.334	Solavetivone	LC-MS; 2D GC-MS	Terpenoid
**12**	389.181	738	C_18_H_30_O_9_	390.425	(−)-11-Hydroxy-9,10-dihydrojasmonic acid 11-beta-D-glucoside	LC-MS	Signaling
**13**	337.09	570.6	C_15_H_20_O_5_	280.316	8′-Hydroxyabscisate	LC-MS	Signaling
**14**	295.1142	774.6	C_12_H_18_O_4_	226.268	epi-4′-Hydroxyjasmonic acid	LC-MS	Signaling
**15**	211.1321	597.6	C_12_H_18_O_3_	210.269	Jasmonic acid	LC-MS	Signaling
**16**	299.078	162	C_13_H_16_O_8_	300.261	Salicylate 2-O-beta-D-glucoside	LC-MS	Signaling
**17**	385.116	401.4	C_17_H_22_O_10_	386.35	1-O-Sinapoyl-beta-D-glucose	LC-MS	Phenylprop
**18**	195.066	604.2	C_10_H_12_O_4_	196.199	2′.6′-Dimethoxy-4′-hydroxyacetophenone	LC-MS	Phenylprop
**19**	319.083	649.2	C_16_H_16_O_7_	320.294	4-Coumaroylshikimate	LC-MS	Phenylprop
**20**	177.055	601.8	C_10_H_8_O_3_	176.168	4-Methylumbelliferone	LC-MS	Phenylprop
**21**	351.071	444.6	C_16_H_16_O_9_	352.292	4-Methylumbelliferone glucuronide	LC-MS	Phenylprop
**22**	337.0925	570.6	C_16_H_18_O_8_	338.309	4-p-Coumaroylquinic acid	LC-MS	Phenylprop
**23**	341.088	349.2	C_15_H_18_O_9_	342.298	Caffeic acid 3-glucoside; 1-Caffeoyl-beta-D-glucose	LC-MS	Phenylprop
**24**	355.102	459.6	C_16_H_18_O_9_	354.308	Caffeoylquinate	LC-MS	Phenylprop
**25**	335.077	559.8	C_16_H_16_O_8_	336.293	Caffeoylshikimate	LC-MS	Phenylprop
**26**	312.123	780	C_17_H_17_NO_2_	267.322	Cinnamoyltyramine	LC-MS	Phenylprop
**27**	181.086	492	C_10_H_12_O_3_	180.2	Coniferyl alcohol	LC-MS	Phenylprop
**28**	147.044	570	C_9_H_6_O_2_	146.142	Coumarin	LC-MS	Phenylprop
**29**	515.117	748.8	C_25_H_24_O_12_	516.45	Dicaffeoylquinic acid	LC-MS	Phenylprop
**30**	469.1107	748.8	C_21_H_22_O_8_	402.394	Flavanone 7-O-beta-D-glucoside	LC-MS	Phenylprop
**31**	193.051	699.6	C_10_H_10_O_4_	194.184	Isoferulic acid	LC-MS	Phenylprop
**32**	251.151	445.2	C_12_H_18_N_4_O_2_	250.297	N-caffeoylputrescine	LC-MS	Phenylprop
**33**	391.1	376.8	C_17_H_20_O_9_	368.335	O-Feruloylquinate	LC-MS	Phenylprop
**34**	191.057	570.6	C_7_H_12_O_6_	192.166	Quinate	LC-MS	Phenylprop
**35**	193.049	384	C_10_H_8_O_4_	192.168	Scopoletin	LC-MS	Phenylprop
**36**	173.046	444.6	C_7_H_10_O_5_	174.151	Shikimate	LC-MS	Phenylprop
**37**	415.124	522.6	C_17_H_22_O_9_	370.351	Sinapaldehyde glucoside	LC-MS	Phenylprop
**38**	225.077	492	C_11_H_12_O_5_	224.209	Sinapate	LC-MS	Phenylprop
**39**	342.134	796.8	C_19_H_21_NO_5_	343.373	Sinapoyltyramine	LC-MS	Phenylprop
**40**	373.148	784.2	C_17_H_24_O_9_	372.367	Syringin	LC-MS	Phenylprop
**41**	355.103	642.6	C_15_H_18_O_7_	310.299	trans-Cinnamoyl beta-D-glucoside	LC-MS	Phenylprop
**42**	195.065	586.2	C_8_H_14_O_4_	174.194	Octadecanoic acid/suberic acid	LC-MS; 2D GC-MS	Fatty acid

* Identification methods/platforms: 1D GC-MS = GC-EI-MS; 2D GC-MS = GC×GC-TOF-MS; LC-MS = UHPLC-HDMS.

** The 1D proton NMR analyses of DLLME samples indicated differential changes in spectral region 3–5.8 ppm, which could be resonance from sesquiterpenoids (rishitin and capsidiol).

For GC×GC-TOF-MS analyses, the Chroma-TOF software was used for automated data analysis from preprocessing to the compound identification step. The software, with its peak finding and deconvolution algorithms features, detects and extracts peaks with their “pure” mass spectra. ChromaTOF software identified compounds by comparing the extracted mass spectra to the NIST mass spectral library. A peak table was then generated from the library search and includes compound names, empirical formulae, Rt (in both dimensions), peak area, CAS numbers, mass spectral match factors and formula weights. The search results (the generated peak table) were evaluated based on a similarity match factor. The latter describes how well the library hit matches the peak when using all masses, and the values above 800 indicate the best fit [83]. In this study, the annotated compounds (similarity factor ≥800) from GC×GC-TOF-MS analyses include terpenoids and phytosterols (Table 1 and Table S1).

### UHPLC-MS Based Annotation of Mixed Polarity Compounds: In-depth Analysis of Metabolomic Data

The variation evidenced by visual inspection of UHPLC-MS BPI chromatograms and explained by PCA modelling of UHPLC-MS data revealed differential changes in intra-cellular metabolite profiles, reflecting thus the response of tobacco cells to ergosterol treatment as a reprogramming of the metabolome [33]. In order to complement the view provided by PCA modelling and closely detect discrimination between ergosterol-treated samples and non-treated controls, a supervised model, OPLS-DA was used (for both ESI positive and negative data, from both DLLME and methanol, ME, samples). OPLS-DA differs from PCA by addition of grouping variables that indicate in which class the samples belong. Where PCA modelling is a descriptive method, OPLS-DA method is an explicative/predictive analysis. The latter facilitates the identification of the metabolite ions responsible for the discrimination between groups. OPLS-DA models data according to *a priori* class information (such as treated *vs.* non-treated) assigned to samples before the analysis; and it is a suitable tool to extract information on changes/differences in the molecular composition of samples under study [14]. OPLS-DA modelling of control and 300 nM ergosterol-treated (incubated for 18 h) samples was performed, to separate multivariate relationships into predictive variation (related to ergosterol treatment) and orthogonal variation (unrelated to ergosterol treatment). Analysis of variance testing of cross-validated predictive residuals (CV-ANOVA), a diagnostic tool, was used to assess the reliability of the obtained OPLS models. A *p*-value that is lower than 0.05 normally indicates a significant model [84], [85].

For DLLME samples, both ESI positive and negative data, the calculated OPLS-DA models were highly significant with a *p*-value of 0.0006 (ESI positive) and 0.005 (ESI negative), and comprised one predictive and three orthogonal components, with a reasonable fit to the data (R^2^X ≥0.714, R^2^Y = 0.99 and Q^2^≥0.817). For ESI positive data, 27% of variation in the computed OPLS-DA model was due to ergosterol treatment whereas for ESI negative data, only 22% of variation was related to ergosterol treatment. In both cases, about 50% of variation in the model was orthogonal variation. For ME samples, ESI positive data, the calculated OPLS-DA model was highly significant with a *p*-value of 8.8×10^−12^, and comprised one predictive and one orthogonal component, with a reasonable fit to the data (R^2^X = 0.62, R^2^Y = 0.996 and Q^2^ = 0.985). 55% of variation in the computed OPLS-DA model was due to ergosterol treatment and 7% was orthogonal variation. For ME samples, ESI negative data, the calculated OPLS-DA model was highly significant with a *p*-value of 9.2×10^−7^, and comprised one predictive and two orthogonal component, with a reasonable fit to the data (R^2^X = 0.645, R^2^Y = 0.993 and Q^2^ = 0.955). 23% of variation in the computed OPLS-DA model was due to ergosterol treatment and 41% was orthogonal variation.

**Figure 6:**
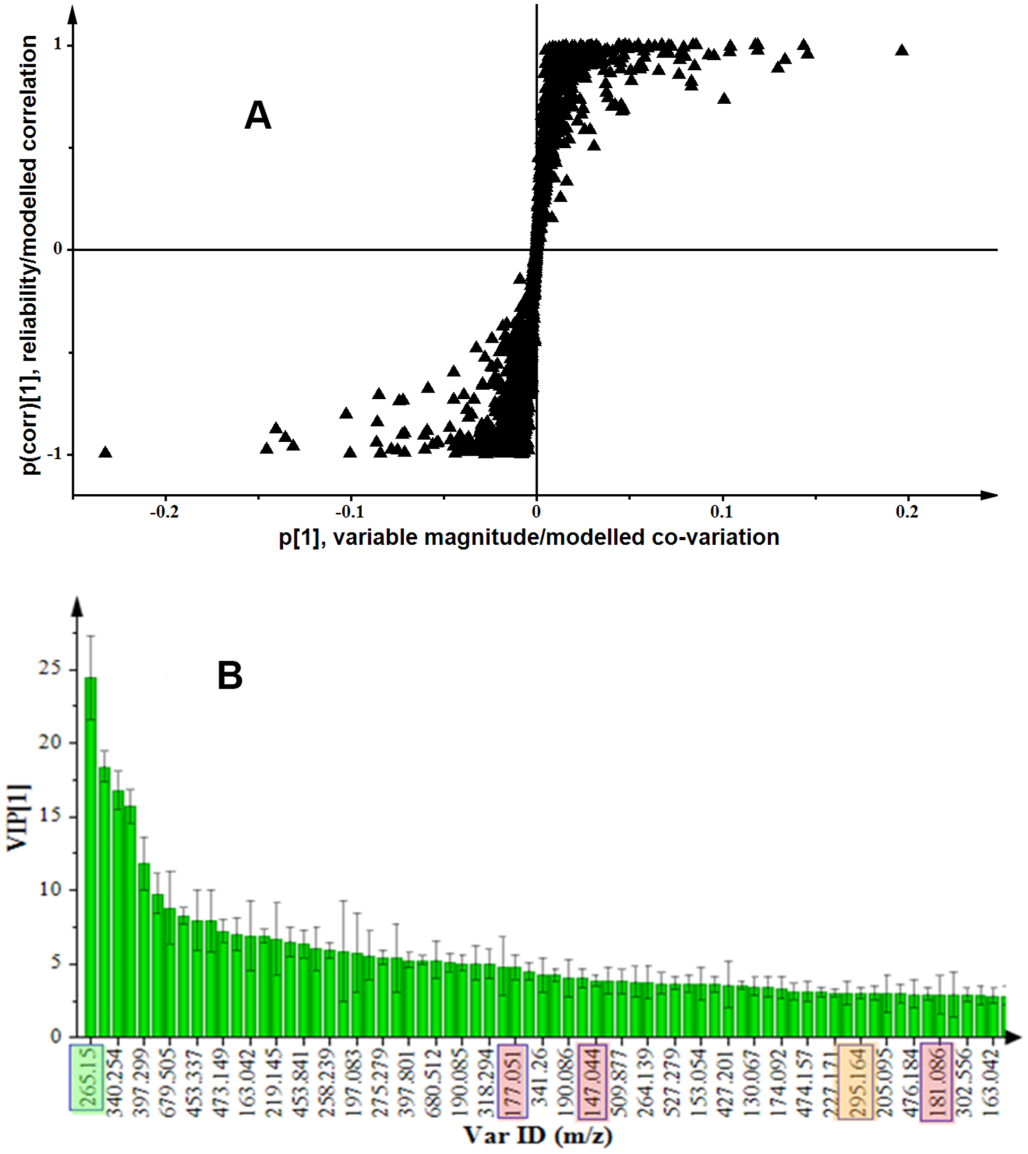
OPLS-DA modelling of data from ergosterol-treated cells. (**A**) S-plot of an OPLS-DA model of control vs. 300 nM ergosterol-treated sample extracts (ME/UHPLC-MS data, ESI positive). The *x*-axis is the modelled covariation (variable magnitude) and the *y*-axis is the loading vector of the predictive component (modelled correlation/reliability). The mass ions in the upper right quadrant of S-plot are positively related to the ergosterol treatment (these include ions *m/z* 265.15, 177.051, 147.044 and 181.086), while those in the lower left quadrant are negatively related to the treatment. (**B**) A variable importance in projection (VIP) plot for an OPLS-DA model of 300 nM-treated samples. The VIP plot indicates for instance that the mass ions *m/z* 256.15, 177.051, 147.044, 295.164 and 181.086 (identified as deoxy-capsidol, methyllumbeliferone, coumarin, epi-hydroxyjasmonic acid and coniferyl alcohol, respectively, Table 1) were accountable for the significant separation in the model as their VIP scores were significantly greater than 1.0.

The evaluation of OPLS-DA loading S-plots (Figure 6A) permitted the extraction of statistically and potentially biochemically significant mass ions (metabolites/bio-markers) in the samples. These mass ions, related to ergosterol treatment, were selected based on their contribution to the model (*x*-axis, modelled covariation) and reliability (*y*-axis, modelled correlation). An ideal statistically significant variable (mass ion, in this case) has high covariation (magnitude) and high reliability, *i.e.* smaller risk for spurious correlations [48], [54]: |*p* [1]| ≥0.05 and |*p*(corr)| ≥0.5, respectively, in this study. Furthermore, the significance of the selected mass ions (variables) in OPLS-DA models was also assessed using the variable importance in projection (VIP) (Figure 6B). The VIP plot summarises the importance of the variables both to explain X and to correlate to Y. The VIP score is a critically important check on the selection of significant ions/variables in a complex data set from metabolomics. The higher the VIP value (exceeding 1.0) the more significant is the ion/variable in the complex analysis in comparing difference between two or more groups [86]–[88]. These mass ions are presented in Table 1 (VIP>1.0) and Table S1 (VIP<1.0), together with their annotations/putative identities.

The selected mass ions from S- and VIP plots were annotated using the Taverna workbench (www.taverna.org.uk) for PUTMEDID_LCMS Metabolite ID Workflows. The Taverna workflows allow for integrated, automated and high-throughput annotation and tentative metabolite identification from ESI-LC-MS metabolic data. The workflows consist of correlation analysis, metabolic feature annotation and metabolite annotation [89].

### Ergosterol Induces Metabolic Reprogramming That Involves Terpenoid and Phenylpropanoid Pathways in Tobacco, Leading to the Biosynthesis of Defence-Related Secondary Metabolites

**Figure 7:**
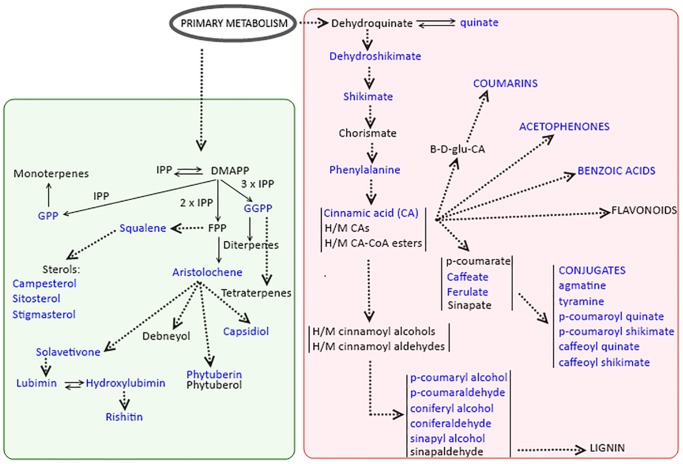
The ergosterol-induced metabolic reprogramming in *N. tabacum* cells. The fungal derived MAMP, ergosterol, induced an intra-cellular metabolic reprogramming that involves terpenoid and phenylpropanoid pathways and their branches, indicated on this diagram. IPP, isopentenyl diphosphate DMAPP, dimethylallyl diphosphate; GGPP, geranylgeranyl diphosphate; FPP, farnesyl diphosphate; H/M CA, hydroxylated/methoxylated cinnamic acids. Metabolites shown in blue have been tentatively identified in this study (Table 1 and Table S1).

For metabolomics to be meaningful and successful, raw analytical data should be converted to structurally elucidated compounds/metabolites in order to provide biological knowledge about the system under investigation [12]. The visual inspection of chromatograms/NMR-spectra and PCA-modelling evidently suggest that the fungal derived MAMP, ergosterol, induced a metabolic reprogramming in tobacco cells that involves a wide range of intracellular secondary metabolites (from thermally stable non-polar metabolites to the thermally labile, semi-polar metabolites, with different molecular weights and chemical structures). Furthermore, translating the observed ergosterol-induced metabolic changes into structurally elucidated metabolites demonstrated that this cellular reprogramming involves a number of metabolic pathways, such as the terpenoid- and phenylpropanoid pathways and their branches. Although no fluxomics analyses were conducted in this study, the annotation of secondary metabolites that are positively or negatively correlated with the treatment, sufficiently suggests dynamic intra- and interrelations among different metabolic pathways (Table 1; Table S1; Figure 7).

The ergosterol-induced components of the terpenoid pathway include the bicyclic sesquiterpenoids (capsidiol, lubimin, phytuberin, rishitin and solavetivone) and their biosynthetic precursor, aristolochene; sesquiterpenoid derivatives (such as deoxy-capsidiol and hydroxylubimin); farnesol, an acyclic sesquiterpene; other precursors in the terpenoids pathway (geranyl diphosphate, GPP) as well as phytosterols (campesterol, sitosterol and stigmasterol) and their precursor, squalene (Table 1; Table S1; Figure 7). These bicyclic sesquiterpenoid metabolites are found in plants within the *Solanaceae*, and are correlated with the defence responses to invading pathogens [90], [91]. Furthermore, farnesol could be conceived of as being involved in regulating the rate of formation of other defence-related terpenoids [92].

The terpenoids are generally synthesized from the isomeric 5-carbon building block molecules, isopentenyl diphosphate (IPP) and dimethylallyl diphosphate (DMAPP), the products of two independent pathways in plants; the mevalonate (MVA) pathway operating in the cytosol and the 2-C-methyl erythritol 4-phosphate (MEP) pathway in plastids [93]–[96]. A series of enzyme-catalysed condensation reactions of IPP and DMAPP molecules leads to the biosynthesis of farnesyl diphosphate (FPP), a 15-carbon molecule. FPP is further converted into a sesquiterpene, 5-*epi*-aristolochene, by the action of a sesquiterpene synthase. Sesquiterpenoids are subsequently enzymatically derived from this common precursor [94], [97]–[99].

The five sesquiterpenoids annotated in this study (also in [33]) have been reported to accumulate in plant cell suspension cultures or tissues challenged by fungal elicitors or by viral infection, thereby providing an antimicrobial and fungitoxic environment [90], [100], [101]. These biotic stress-induced sesquiterpenoids are known phytoalexins [91], [98], [101], referring to host-synthesised low molecular weight compounds with protective properties of which *de novo* biosynthesis and accumulation are induced in plants following biotic or abiotic stress. The phytoalexin response is an effective part of the multi-component stress resistance mechanism in plants [101]–[103].

**Figure 8:**
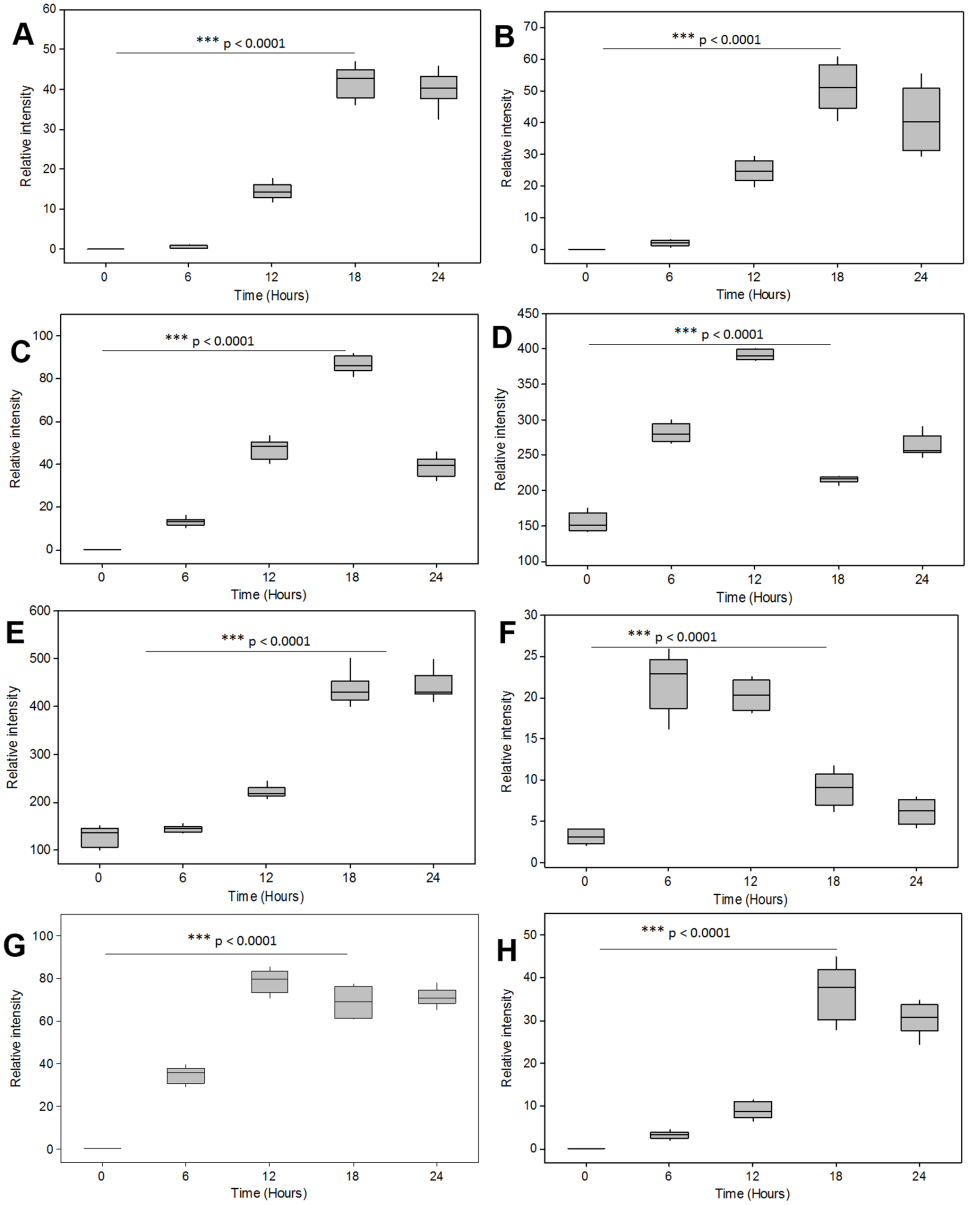
The quantitative, time-dependent, responses of some of the annotated compounds from different metabolite classes: (**A**–**C**) sesquiterpenoid phytoalexins (rishitin, solavetivone and phytuberin), (**D**–**E**) sterols (squalene and stigmasterol) and (**F**–**H**) phenolics (shikimate, caffeoyl shikimate and coniferyl alcohol). This relative quantification is based on their respective peak areas estimated from peak integration. The quantitative estimation shows a time-dependent biosynthesis of sesquiterpenoids, sterols and phenylpropanoids from cells treated with 300 nM ergosterol, reaching a maximum at 18 h post-treatment (N-ANOVA t-test, *p*<0.0001).

Furthermore, a relative quantitative analysis of selected sesquiterpenoids (rishitin, solavetivone and phytuberin) (Figure 8-A, -B and -C, respectively) indicated that accumulation of these phytoalexins was significantly detectable from 12 h post-treatment, reached a maximum at 18 h (*p*<0.0001) and at 24 h their levels started to decrease. It is possible that from 24 h, these induced metabolites are either secreted to the extracellular milieu or are degraded. After the infection or stress has been contained or accommodated, the plant normally degrades antimicrobial compounds in enzyme-catalysed reactions, returning their levels to pre-infection or pre-stress concentrations [77].

Some signaling molecules and –derivatives/conjugates were also tentatively identified. Plant hormone signaling pathways related to defence are not isolated but rather interconnected through complex regulatory networks. The type of interactions and plant responses to stresses vary depending on the pathosystem as well as the time, quantity and the tissue where the hormones are produced. To understand how plants coordinate multiple hormonal components in response to various developmental and environmental cues is a major challenge [104]. The annotated hormones include jasmonic acid (JA) and its hydroxylated derivative; salicylate (SA - a known defence signal) glucoside; methylsalicylate (MeSA); as well as abscisate (ABA) and hydroxyabscisate, an ABA degradation product (Table 1, Table S1). Studies have shown that the activity of ABA-8′-hydroxylase is up-regulated by ABA accumulation and stress [105]. ABA is an isoprenoid plant hormone (C_15_-molecule) synthesised in the plastidial MEP pathway [106], [107], unlike other structurally related C_15_-terpenoids (sesquiterpenoids) that are synthesised from the cytosolic MVA pathway. ABA is vitally involved in the regulation of various aspects of plant growth and development. Furthermore, ABA regulates plant defence responses negatively or positively, depending on the type of plant : pathogen interaction or the nature of abiotic stress. The role of ABA in plant resistance appears to be complex and the exact molecular mechanism of this phytohormone in defence responses is still unclear [104], [108]–[113]. The findings from a study conducted on *Nicotiana plumbaginifolia* suggested that even though ABA may not be required *per se* for the biosynthesis of capsidiol (a sesquiterpenoid phytoalexin), this phytohormone is vitally implicated in a stress-response checkpoint to fine-tune the amplification of capsidiol synthesis in challenged plants [114]. Azelaic acid, a mobile molecule that can confer local and systemic resistance via priming of plants to accumulate SA upon infection [115], was also found in extracts from ergosterol-treated cells (Table S1).

The MS-based annotation of treatment-correlated metabolites indicates further that the ergosterol-induced metabolic reprogramming involved also the phenylpropanoid pathway and its branches (Figure 7; Table 1; Table S1). Coumarins, acetophenones and benzoic acids as well as a range of lignin precursor molecules were found. These metabolites are known in the context of plant stress - and defence responses [68]. Of special interest is the different conjugates of cinnamic acid derivatives that were annotated as being positively correlated with the ergosterol-induced responses. A close examination of some of the annotated phenylpropanoid compounds indicated also a significant time-dependent accumulation of these compounds (*p*<0.0001) (Figures 8-F to -H). The hydroxylated and methoxylated cinnamoyl-tyramine conjugates have been reported to accumulate in response to priming with bacterial lipopolysaccharides (LPS) and pathogen inoculation. These compounds have antimicrobial activity and can also be cross-linked into the plant wall to strengthen it and can thus play a role in the resistance response of plants [20], [116]. The results indicate that ergosterol affects a wide activation of the shikimate - and phenylpropanoid - linked defence pathways, the products of which may subsequently lead to an antimicrobial environment *in vivo.*

The fatty acids (FAs, saturated and unsaturated) annotated from GC-MS and GC×GC-TOF-MS analyses (Table 1; Table S1), include n-hexadecanoic acid (16∶0, palmitic acid), octadecanoic acid (18∶0, stearic acid) and 9,12-octadecadienoic acid (Z,Z) (18∶2, linoleic acid). Recent studies have demonstrated that FAs and their breakdown products participate actively and directly in various modes of plant defences, such as remodelling cell membrane fluidity and modulating defence gene expression. Both C_16_ and C_18_ FAs are involved in regulating basal, effector-triggered, and systemic immunity in plants. It has been shown that oleic acid (18∶1) and linoleic acid (18∶2) play significant roles in the activation of NADPH oxidase, resulting in the production of reactive oxygen species (ROS), both components of the plant defence reaction [117]–[119].

Phytosterols (campesterol, stigmasterol and β-sitosterol) and their precursor, squalene were among the compounds annotated using GC×GC-TOF-MS (Table 1; Table S1). Relative quantification indicated that squalene accumulated significantly 12 h post-treatment (*p*<0.0001) and the level decreased as that of stigmasterol reached a maxium at 18 h (*p*<0.0001) (Figures 8-D and –E). This observed variation of the detected phytosterols’ level suggests a time-dependent response to ergosterol treatment. Although the sterols are constitutive major components of the cell membrane, recent discoveries (in *N. benthamiana* and *Arabidopsis thaliana* systems) have demonstrated that plants alter sterol biosynthesis (particularly stigmasterol and β-sitosterol) upon pathogen attack so as to restrict nutrient transfer from the cytosol to the apoplastic space [120]. Furthermore, studies have pointed out changes in expression levels of *A. thaliana* C24 methyltransferases At1g20330 and At5g13710 genes following the treatment with LPS [121]. These genes encode enzymes that control carbon flux into sterol biosynthesis, influencing subsequently membrane permeability. A pathogen-induced alteration in the stigmasterol/β-sitosterol ratio can influence the physicochemical properties of membrane microdomains and thereby modulate plant defence signaling [122].

Both sesquiterpenoids and sterols are biosynthesised *via* the terpenoid pathway. Studies have shown that the position of FPP in the terpenoid biosynthetic pathway is an important and potential regulatory branch point of sesquiterpenoid biosynthesis. Under normal conditions, the FPP is channelled toward the biosynthesis of sterol and prenyl-lipid moieties [94], [99]. Moreover, the enhanced expression of sesquiterpene cyclase, a key enzyme in the biosynthesis of sesquiterpenoids in response to treatment with tobacco mosaic virus or cell wall fragments from *Phytophthora* species, was accompanied by the suppression of squalene synthase activity in tobacco and potato cells/tissues [32], [94], [123], [124]. Thus, the detection of both sesquiterpenoids and phytosterols in response to ergosterol suggests a complex regulation of the terpenoid pathway, permitting the production of both the sesquiterpenoid phytoalexins (fungitoxicity) and the increase of the levels of phytosterols (to restrict cell membrane permeability). However, further quantitative analyses and molecular-based studies are needed to corroborate these observations and unfold the molecular mechanisms involved in the complexity of regulatory network structures.

## Conclusion

Ergosterol acts as a MAMP molecule in tobacco and tomato plants, triggering a defence response characterised by the elicitation of the oxidative burst, the production of ROS [31], [32], alkalinisation of the external milieu and mobilisation of cytosolic calcium [30], [31], [125], [126]. Furthermore, ergosterol induces expression of some genes encoding pathogenesis-related proteins (PR1, PR3, and PR5) and enzymes participating in the defence response such as phenylalanine-ammonia lyase and sesquiterpene cyclase [32]. This ergosterol-induced defence response, a cellular reprogramming, is reflected also by dynamic and differential changes to the metabolome as demonstrated by our results.

The presence of the five bicyclic sesquiterpenoids (phytuberin, solavetivone, capsidiol, lubimin and rishitin) in ergosterol-elicited tobacco cells and other annotated metabolites (abscisic acid, fatty acids, phytosterols and metabolites of shikimate-phenylpropanoid pathways) indicates that the changes in the metabolome, demonstrated by chromatographic/spectral analyses and PCA-explained variations, are associated with a defensive function (‘defensome’) in response to elicitation by ergosterol as a MAMP molecule. Ergosterol induced a complex and dynamic activation of the terpenoid, shikimate-phenylpropanoid and FA pathways leading to *de novo* biosynthesis of sesquiterpenoids phytoalexins and alteration in signaling molecules (JA, SA, MeSA, ABA, azelaic acid), FAs, phenylpropanoid-metabolies and phytosterols involved in various modes of defence responses.

This ergosterol-induced metabolic reprogramming is dynamic and largely complex as it involves changes in different metabolic pathways which points to interconnected alterations in metabolic networks that are functionally correlated. The groups of identified compounds serve not only as a base in the search of novel defence compounds, but also as signatory bio-markers for the characterisation of the plants’ defensive state. This study identified mainly sesquiterpenoids, phytosterols, phenylpropanoid-metabolites (and conjugates/derivatives) and fatty acids, hence further metabolic network investigations are needed to unfold these intra- and interrelationships among different metabolic pathways involved in the ergosterol-induced response and to provide insights into the molecular mechanisms tangled in the complexity of regulatory network structures.

## Supporting Information

Figure S1 **ESI positive BPI MS chromatograms of methanol extracts separated by UHPLC. (A)** Concentration study. The chromatograms show treatment-related variations. ME samples of the cells treated with different ergosterol concentrations (from bottom to top: 0, 50, 150, 300 and 1000 nM) and incubated for 18 h. **(B)** Time study. The chromatograms show some time-dependent treatment-related variations. ME samples of cells treated with 300 nM ergosterol and incubated for different time periods (from bottom to top: 0, 6, 12, 18 and 24 h T) and the top chromatogram is a non-treated sample incubated for 24 h (24 h NT). Visual inspection of these compared mass chromatograms (in both sets: concentration- and time study) indicate differential changes in detected mass ions, reflecting ergosterol-induced changes (concentration- and time-dependent) in the (ME-) metabolite profiles of the tobacco cells.
(TIF)

Figure S2 **PCA scores plot of methanol extracts separated by UHPLC-MS in ESI positive mode. (A)** Concentration study. ME samples of the cells treated with 0 nM (control), 50 nM, 150 nM, 300 nM, and 1000 nM ergosterol, and incubated for 18 h. A PCA-four-component model was calculated and explained 77.3% of the variance [*R^2^X*(cum) of 0.772 and *Q^2^*(cum) of 0.522]. Using the first two principal components (PC1 and PC2, explaining 69.5% of the variance, with 95% confidence level) for a scores plot, the tobacco samples were found to be differentially clustered into five groups corresponding to different ergosterol treatments (0–1000 nM) with no significant intra-group variation. The clusters corresponding to 150 nM, 300 nM and 1000 nM are seen to group close to each other. The control (non-treated) and 50 nM samples are clustered close to each other and significantly separated from the rest of the treatments. This scores plot evidenced a clear dosage-dependent variation, suggesting also a similarity and less variability in metabolite content of 0 nM and 50 nM samples. **(B)** Time study. ME samples of the cells treated with 300 nM ergosterol, and incubated for incubated for different time periods (0 h T - 24 h T) and a non-treated sample incubated for 24 h (24 h NT). A two-component model was calculated and explained 68.7% of the variance [*R^2^X*(cum) of 0.688 and *Q^2^*(cum) of 0.580]. A PCA scores plot (95% confidence) was constructed using PC1 and PC2, showing samples differentially clustered into different groups: the 6 h- incubated samples formed a separate cluster, and the rest of the samples clustered separately but closer to each other. The scores plot shows a time-dependent variation.
(TIF)

Figure S3 **OPLS-DA model of non-treated vs. 300 nM ergosterol-treated samples (ME/UHPLC-MS data, ESI negative).** The calculated model had one predictive and one orthogonal components, and explained 81.7% of the variation [68.7% treatment-related variation (predictive) and 13.0% orthogonal variation]. **(A)** The loading S-plot of the calculated model. The *x*-axis is the modelled covariation (variable magnitude) and the *y*-axis is the loading vector of the predictive component (modelled correlation/reliability). The mass ions in the upper right quadrant of S-plot are positively related to the ergosterol treatment, while those in the lower left quadrant are negatively related to the treatment. **(B)** A variable importance in projection (VIP) plot for an OPLS-DA model of 300 nM-treated samples. The VIP plot indicates for instance that the mass ions *m/z* 351.069, 335.077 and 337.09 (identified as methylumbeliferone glucuronide, caffeoylshikimate and hydroxyabscisate, respectively, Table 1 and Table S1) were accountable for the significant separation in the model as their VIP scores were significantly greater than 1.0.
(TIF)

Table S1 Some of the tentatively identified (M-level 2) metabolites (with VIP<1.0) from ergosterol-treated tobacco cells.
(DOCX)
