# Supplementary figures and images for: Multi-Platform Metabolomic Analyses of Ergosterol-Induced Dynamic Changes in Nicotiana tabacum Cells
Source: PLoS One. 2014 Jan 31;9(1):e87846. doi: 10.1371/journal.pone.0087846 (PMC3909234; doi:10.1371/journal.pone.0087846)

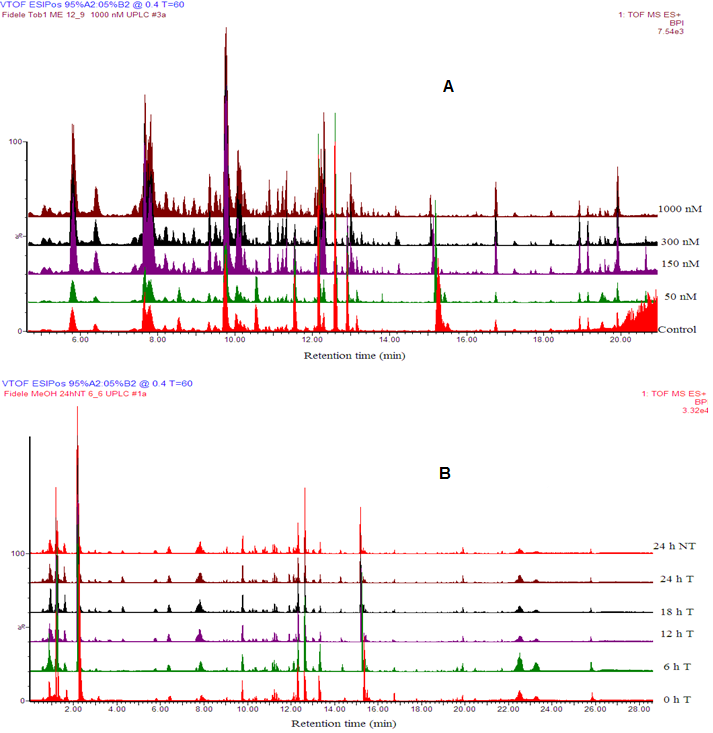

Supplement: Figure S1 — ESI positive BPI MS chromatograms of methanol extracts separated by UHPLC. (A) Concentration study. The chromatograms show treatment-related variations. ME samples of the cells treated with different ergosterol concentrations (from bottom to top: 0, 50, 150, 300 and 1000 nM) and incubated for 18 h. (B) Time study. The chromatograms show some time-dependent treatment-related variations. ME samples of cells treated with 300 nM ergosterol and incubated for different time periods (from bottom to top: 0, 6, 12, 18 and 24 h T) and the top chromatogram is a non-treated sample incubated for 24 h (24 h NT). Visual inspection of these compared mass chromatograms (in both sets: concentration- and time study) indicate differential changes in detected mass ions, reflecting ergosterol-induced changes (concentration- and time-dependent) in the (ME-) metabolite profiles of the tobacco cells. (TIF) [file pone.0087846.s001.tif]

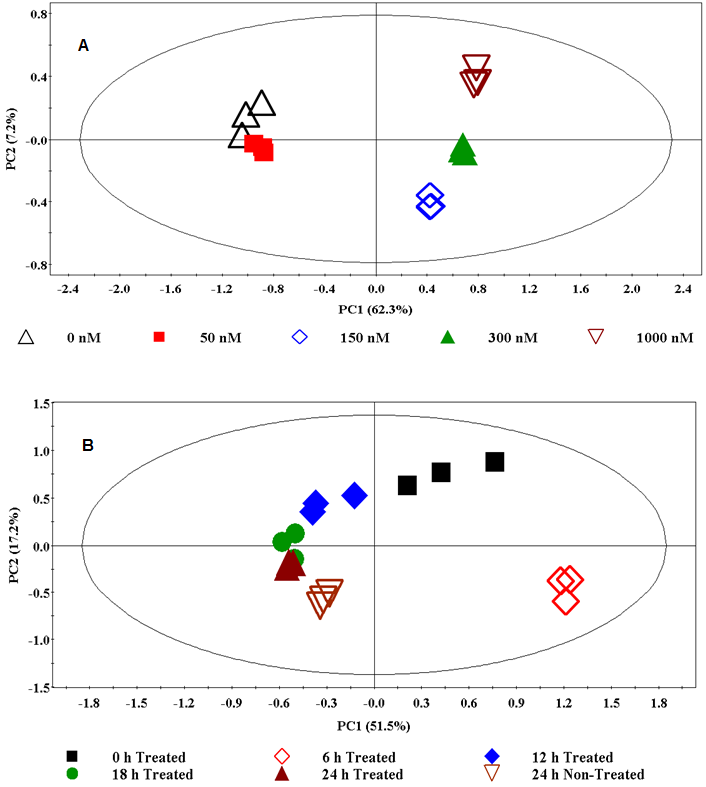

Supplement: Figure S2 — PCA scores plot of methanol extracts separated by UHPLC-MS in ESI positive mode. (A) Concentration study. ME samples of the cells treated with 0 nM (control), 50 nM, 150 nM, 300 nM, and 1000 nM ergosterol, and incubated for 18 h. A PCA-four-component model was calculated and explained 77.3% of the variance [R2X(cum) of 0.772 and Q2(cum) of 0.522]. Using the first two principal components (PC1 and PC2, explaining 69.5% of the variance, with 95% confidence level) for a scores plot, the tobacco samples were found to be differentially clustered into five groups corresponding to different ergosterol treatments (0–1000 nM) with no significant intra-group variation. The clusters corresponding to 150 nM, 300 nM and 1000 nM are seen to group close to each other. The control (non-treated) and 50 nM samples are clustered close to each other and significantly separated from the rest of the treatments. This scores plot evidenced a clear dosage-dependent variation, suggesting also a similarity and less variability in metabolite content of 0 nM and 50 nM samples. (B) Time study. ME samples of the cells treated with 300 nM ergosterol, and incubated for incubated for different time periods (0 h T - 24 h T) and a non-treated sample incubated for 24 h (24 h NT). A two-component model was calculated and explained 68.7% of the variance [R2X(cum) of 0.688 and Q2(cum) of 0.580]. A PCA scores plot (95% confidence) was constructed using PC1 and PC2, showing samples differentially clustered into different groups: the 6 h- incubated samples formed a separate cluster, and the rest of the samples clustered separately but closer to each other. The scores plot shows a time-dependent variation. (TIF) [file pone.0087846.s002.tif]

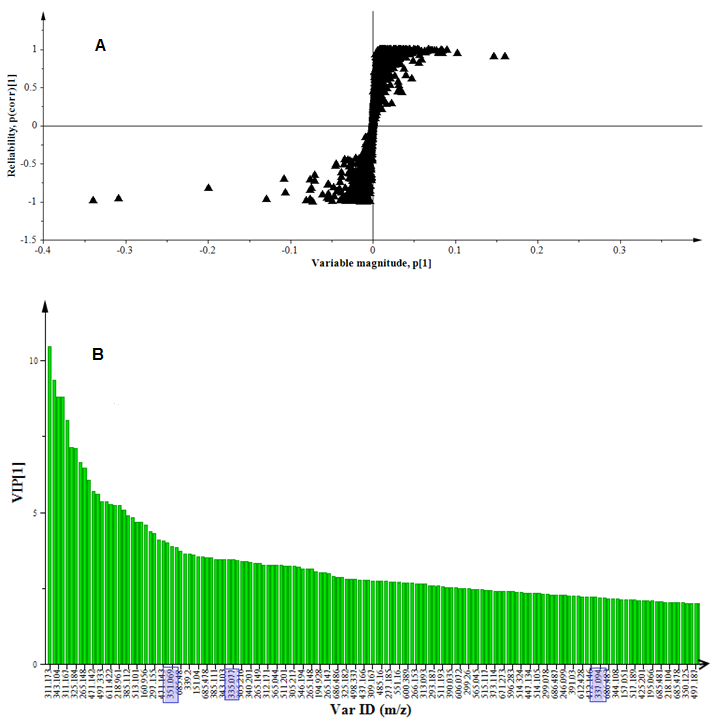

Supplement: Figure S3 — OPLS-DA model of non-treated vs. 300 nM ergosterol-treated samples (ME/UHPLC-MS data, ESI negative). The calculated model had one predictive and one orthogonal components, and explained 81.7% of the variation [68.7% treatment-related variation (predictive) and 13.0% orthogonal variation]. (A) The loading S-plot of the calculated model. The x-axis is the modelled covariation (variable magnitude) and the y-axis is the loading vector of the predictive component (modelled correlation/reliability). The mass ions in the upper right quadrant of S-plot are positively related to the ergosterol treatment, while those in the lower left quadrant are negatively related to the treatment. (B) A variable importance in projection (VIP) plot for an OPLS-DA model of 300 nM-treated samples. The VIP plot indicates for instance that the mass ions m/z 351.069, 335.077 and 337.09 (identified as methylumbeliferone glucuronide, caffeoylshikimate and hydroxyabscisate, respectively, Table 1 and Table S1) were accountable for the significant separation in the model as their VIP scores were significantly greater than 1.0. (TIF) [file pone.0087846.s003.tif]
