# Supplementary material for: Multi-Platform Metabolomic Analyses of Ergosterol-Induced Dynamic Changes in Nicotiana tabacum Cells
Source: PLoS One. 2014 Jan 31;9(1):e87846. doi: 10.1371/journal.pone.0087846 (PMC3909234; doi:10.1371/journal.pone.0087846)
Supplement: Table S1 — Some of the tentatively identified (M-level 2) metabolites (with VIP<1.0) from ergosterol-treated tobacco cells. (DOCX) [file pone.0087846.s004.docx]

**Table S1**: Some of the tentatively identified metabolites (with VIP < 1.0) from ergosterol-treated tobacco cells

| # | *m/z* | RT  (s) | MF | MW (ave) | Compound ID name | Platform | Metabolite  category |
| --- | --- | --- | --- | --- | --- | --- | --- |
| 1 | 263.238 | 1257.6 | C_18_H_30_O | 262.43 | (E,E)-Farnesylacetone | LC-MS_DLLME | Terpenoid |
| 2 | 322 | 1060; 2.23 | C_21_H_38_O_2_ | 322 | 11,14-Eicosadienoic acid, methyl ester | GCxGC-TOFMS | Terpenoid |
| 3 | 206 | 685; 1.88 | C_14_H_22_O | 206 | 11-nor-8-Drimen-7-one (isonordrimenone) | GCxGC-TOFMS + 1D GC-MS | Terpenoid |
| 4 | 489.3584 | 1338 | C_30_H_48_O_5_ | 488.699 | Bayogenin | LC-MS_DLLME | Terpenoid |
| 5 | 400 | 1585; 2.62 | C_28_H_48_O | 400 | Campesterol | GCxGC-TOFMS | Terpenoid |
| 6 | 195.1389 | 1036.2 | C_12_H_20_O_2_ | 196.286 | Geranyl acetate | LC-MS_DLLME | Terpenoid |
| 7 | 330.0891 | 502.2 | C_10_H_20_O_7_P_2_ | 314.209 | Geranyl diphosphate | LC-MS_ME | Terpenoid |
| 8 | 315.1954 | 1098.6 | C_20_H_28_O_3_ | 316.434 | Gibberellin A12 aldehyde | LC-MS_ME | Terpenoid |
| 9 | 215.1291 | 879.6 | C_10_H_18_O_2_ | 170.248 | Limonene-1,2-diol | LC-MS_ME | Terpenoid |
| 10 | 293.1762 | 1026 | C_17_H_26_O_4_ | 294.385 | Phytuberin | LC-MS_DLLME | Terpenoid |
| 11 | 587.3259 | 662.4 | C_30_H_52_O_7_P_2_ | 586.677 | Presqualene diphosphate | LC-MS_ME | Terpenoid |
| 12 | 410 | 1350; 2.63 | C_30_H_50_ | 410 | Squalene | GCxGC-TOFMS | Terpenoid |
| 13 | 412 | 1595; 2.67 | C_29_H_48_O | 412 | Stigmasterol | GCxGC-TOFMS | Terpenoid |
| 14 | 154 | 828 | C_10_H_18_O | 154 | Linalool | 1D GC-MS | Terpenoid |
| 15 | 414 | 1615; 2.62 | C_29_H_50_O | 414 | β-Sitosterol | GCxGC-TOFMS | Terpenoid |
| 16 | 187.0984 | 710.4 | C_9_H_16_O_4_ | 188.220 | Azelaic acid | LC-MS_DLLME | Signaling |
| 17 | 265.144 | 655.2 | C_15_H_20_O_4_ | 264.316 | Abscisate | LC-MS_ME | Signaling |
| 18 | 151.0406 | 627.6 | C_8_H_8_O_3_ | 152.147 | Methylsalicylate | LC-MS_ME | Signaling |
| 19 | 307.0794 | 337.2 | C_13_H_18_O_7_ | 286.277 | Salicin | LC-MS_ME | Signaling |
| 20 | 374.1429 | 621 | C_16_H_20_O_9_ | 356.324 | 1-O-Feruloyl-beta-D-glucose | LC-MS_ME | Phenylprop and branches |
| 21 | 275.0301 | 912.6 | C_13_H_10_O_4_ | 230.216 | 2,4,6-Trihydroxybenzophenone | LC-MS_ME | Phenylprop and branches |
| 22 | 135.0454 | 917.4 | C_8_H_8_O_2_ | 136.147 | 2-Hydroxyacetophenone | LC-MS_DLLME | Phenylprop and branches |
| 23 | 361.0919 | 570 | C_18_H_16_O_8_ | 360.314 | 3',4',5-Trihydroxy-3,6,7-trimethoxyflavone | LC-MS_ME | Phenylprop and branches |
| 24 | 249.0649 | 627 | C_12_H_14_O_2_ | 190.238 | 3-Dimethylallyl-4-hydroxybenzaldehyde | LC-MS_ME | Phenylprop and branches |
| 25 | 193.0487 | 384 | C_10_H_8_O_4_ | 192.168 | 4-Carboxycinnamic Acid | LC-MS_ME | Phenylprop and branches |
| 26 | 227.1045 | 144 | C_13_H_16_O_2_ | 204.264 | 4'-Hydroxy-3'-prenylacetophenone | LC-MS_ME | Phenylprop and branches |
| 27 | 381.1142 | 459.6 | C_15_H_20_O_7_ | 312.315 | 4-Hydroxycinnamyl alcohol 4-D-glucoside | LC-MS_ME | Phenylprop and branches |
| 28 | 247.057 | 196.8 | C_7_H_14_O_4_ | 162.183 | 4-Methoxycinnamic acid | LC-MS_ME | Phenylprop and branches |
| 29 | 339.1084 | 897.6 | C_16_H_18_O_8_ | 338.309 | 4-p-Coumaroylquinic acid | LC-MS_ME + DLLME | Phenylprop and branches |
| 30 | 344.9985 | 127.8 | C_10_H_13_O_10_P | 324.177 | 5-Enolpyruvyl-shikimate-3-phosphate | LC-MS_ME | Phenylprop and branches |
| 31 | 193.0509 | 627.6 | C_10_H_10_O_4_ | 194.184 | 5-Hydroxyconiferaldehyde | LC-MS_ME | Phenylprop and branches |
| 32 | 195.0659 | 459 | C_10_H_12_O_4_ | 196.199 | 5-Hydroxyconiferyl alcohol | LC-MS_ME | Phenylprop and branches |
| 33 | 197.0805 | 664.8 | C_10_H_12_O_4_ | 196.199 | Acetosyringone | LC-MS_ME + 2D GC-MS | Phenylprop and branches |
| 34 | 210.0776 | 1158 | C_9_H_11_NO_9_ | 277.184 | Aminohydrocinnamic acid | LC-MS_ME | Phenylprop and branches |
| 35 | 116 | 340; 1.5 | C_9_H_8_ | 116 | Benzene,1-ethynyl-2-methyl- | GCxGC-TOFMS | Phenylprop and branches |
| 36 | 167 | 1055; 1.44 | C_8_H_9_NO_3_ | 167 | Benzoic acid amide, 4-hydroxy-3-methoxy- | GCxGC-TOFMS | Phenylprop and branches |
| 37 | 359.1027 | 278.4 | C_19_H_18_N_2_O_4_ | 338.357 | Caffeoylserotonin | LC-MS_ME | Phenylprop and branches |
| 38 | 337.0927 | 727.8 | C_16_H_16_O_8_ | 336.29 | Caffeoylshikimate | LC-MS_ME | Phenylprop and branches |
| 39 | 247.0569 | 223.2 | C_7_H_14_O_4_ | 162.183 | Coniferyl aldehyde | LC-MS_ME | Phenylprop and branches |
| 40 | 193.0513 | 699.6 | C_9_H_8_O_3_ | 164.158 | Coumaraldehyde | LC-MS_ME | Phenylprop and branches |
| 41 | 195.0655 | 604.2 | C_9_H_10_O_2_ | 150.174 | Coumaryl-alcohol | LC-MS_ME | Phenylprop and branches |
| 42 | 171.03 | 129 | C_7_H_8_O_5_ | 172.135 | Dehydroshikimate | LC-MS_ME | Phenylprop and branches |
| 43 | 181.0513 | 559.8 | C_9_H_10_O_4_ | 182.173 | Dihydrocaffeic acid | LC-MS_ME | Phenylprop and branches |
| 44 | 193.0508 | 590.4 | C_10_H_10_O_4_ | 194.184 | Ferulate | LC-MS_ME | Phenylprop and branches |
| 45 | 227.1077 | 975 | C_15_H_14_O_2_ | 226.27 | Flavanol | LC-MS_DLLME | Phenylprop and branches |
| 46 | 393.0582 | 468.6 | C_19_H_14_O_8_ | 370.309 | Flavodic acid | LC-MS_ME pos | Phenylprop and branches |
| 47 | 525.0787 | 1246.8 | C_24_H_22_O_11_ | 486.424 | Flavonol 3-O-(6-O-malonyl-beta-D-glucoside) | LC-MS_DLLME | Phenylprop and branches |
| 48 | 303.0849 | 143.4 | C_16_H_14_O_6_ | 302.278 | Hesperetin | LC-MS_ME | Phenylprop and branches |
| 49 | 210.0782 | 971.4 | C_9_H_11_NO_2_ | 165.189 | L-phenylalanine | LC-MS_ME | Phenylprop and branches |
| 50 | 193.0508 | 590.4 | C_10_H_10_O_4_ | 194.184 | Methyl caffeate | LC-MS_ME | Phenylprop and branches |
| 51 | 312.1251 | 750 | C_18_H_19_NO_4_ | 313.347 | N-Feruloyltyramine | LC-MS_DLLME | Phenylprop and branches |
| 52 | 417.1035 | 502.8 | C_17_H_20_O_12_ | 416.333 | O-Sinapoylglucarate | LC-MS_ME | Phenylprop and branches |
| 53 | 345.12 | 252 | C_19_H_18_N_2_O_3_ | 322.357 | p-Coumaroylserotonin | LC-MS_ME | Phenylprop and branches |
| 54 | 335.0783 | 496.8 | C_15_H_14_O_6_ | 290.268 | p-Coumaroyltriacetate | LC-MS_ME | Phenylprop and branches |
| 55 | 151.112 | 536.4 | C_10_H_14_O | 150.217 | p-Cumic alcohol | LC-MS_ME | Phenylprop and branches |
| 56 | 362.2901 | 1008.6 | C_18_H_39_NO_3_ | 317.507 | Phytosphingosine | LC-MS_DLLME | Phenylprop and branches |
| 57 | 207.066 | 805.2 | C_11_H_10_O_4_ | 206.194 | Scoparone | LC-MS_ME | Phenylprop and branches |
| 58 | 277.008 | 347.4 | C_7_H_11_O_8_P | 254.131 | Shikimate 5-phosphate;Shikimate 3-phosphate | LC-MS_ME | Phenylprop and branches |
| 59 | 341.0859 | 162 | C_15_H_16_O_9_ | 340.282 | Sinapoyl malate | LC-MS_ME | Phenylprop and branches |
| 60 | 211.0971 | 139.2 | C_11_H_14_O_4_ | 210.22 | Sinapyl alcohol | LC-MS_ME | Phenylprop and branches |
| 61 | 341.1015 | 739.8 | C_19_H_18_O_6_ | 342.342 | Tetramethoxyflavone | LC-MS_ME | Phenylprop and branches |
| 62 | 224 | 2083.8; 2.11 | C_15_H_28_O | 224 | (Z)6,(Z)9-Pentadecadien-1-ol | GCxGC-TOFMS | Fatty acid |
| 63 | 311.22 | 1033.8 | C_16_H_32_O_4_ | 288.422 | 10,16-Dihydroxyhexadecanoic acid | LC-MS_DLLME | Fatty acid |
| 64 | 269.213 | 1169.4 | C_16_H_30_O_3_ | 270.407 | 3-Oxo-hexadecanoic acid | LC-MS_DLLME | Fatty acid |
| 65 | 282.2781 | 1168.8 | C_18_H_32_O | 264.446 | 9,12-Octadecadienal | LC-MS_DLLME | Fatty acid |
| 66 | 280 | 2080; 0.77 | C_18_H_32_O_2_ | 280 | 9,12-Octadecadienoic acid (Z,Z)- | GCxGC-TOFMS | Fatty acid |
| 67 | 294 | 1025; 2.18 | C_19_H_34_O_2_ | 294 | 9,12-Octadecadienoic acid, methyl ester | GCxGC-TOFMS | Fatty acid |
| 68 | 270 | 905; 2.33 | C_17_H_34_O_2_ | 270 | Hexadecanoic acid, methyl ester | GCxGC-TOFMS | Fatty acid |
| 69 | 239.2381 | 1549.8 | C_16_H_30_O | 238.408 | Hexadecenal | LC-MS_DLLME | Fatty acid |
| 70 | 256 | 945; 2.33 | C_16_H_32_O_2_ | 256 | n-Hexadecanoic acid | GCxGC-TOFMS + 1D GC-MS | Fatty acid |
| 71 | 305.2248 | 1290.6 | C_18_H_36_O | 268.477 | Octadecanal | LC-MS_DLLME | Fatty acid |
| 72 | 298 | 1015; 2.41 | C_19_H_38_O_2_ | 298 | Octadecanoic acid, methyl ester | GCxGC-TOFMS | Fatty acid |
| 73 | 284.2954 | 1302.6 | C_18_H_34_O | 266.461 | Octadecenal | LC-MS_DLLME | Fatty acid |
| 74 | 228 | 639 | C_14_H_32_O_2_ | 228 | Myristic acid | 1D GC-MS | Fatty acid |
